# Enhanced Degradation of Methyl Orange and Trichloroethylene with PNIPAm-PMMA-Fe/Pd-Functionalized Hollow Fiber Membranes

**DOI:** 10.3390/nano13142041

**Published:** 2023-07-10

**Authors:** Rollie Mills, Cameron Tvrdik, Andrew Lin, Dibakar Bhattacharyya

**Affiliations:** Department of Chemical and Materials Engineering, University of Kentucky, Lexington, KY 40508, USA; rolliegmills@uky.edu (R.M.); cameron.tvrdik@uky.edu (C.T.); andrew.lin@uky.edu (A.L.)

**Keywords:** hollow fiber membrane, methyl orange, trichloroethylene, thermoresponsive, PNIPAm, bimetallic catalysts, water detoxification, zero-valent iron

## Abstract

Trichloroethylene (TCE) is a prominent groundwater pollutant due to its stability, widespread contamination, and negative health effects upon human exposure; thus, an immense need exists for enhanced environmental remediation techniques. Temperature-responsive domains and catalyst incorporation in membrane domains bring significant advantages for toxic organic decontamination. In this study, hollow fiber membranes (HFMs) were functionalized with stimuli-responsive poly-N-isopropylacrylamide (PNIPAm), poly-methyl methacrylate (PMMA), and catalytic zero-valent iron/palladium (Fe/Pd) for heightened reductive degradation of such pollutants, utilizing methyl orange (MO) as a model compound. By utilizing PNIPAm’s transition from hydrophilic to hydrophobic expression above the LCST of 32 °C, increased pollutant diffusion and adsorption to the catalyst active sites were achieved. PNIPAm-PMMA hydrogels exhibited 11.5× and 10.8× higher equilibrium adsorption values for MO and TCE, respectively, when transitioning from 23 °C to 40 °C. With dip-coated PNIPAm-PMMA-functionalized HFMs (weight gain: ~15%) containing Fe/Pd nanoparticles (d_p_~34.8 nm), surface area-normalized rate constants for batch degradation were determined, resulting in a 30% and 420% increase in degradation efficiency above 32 °C for MO and TCE, respectively, due to enhanced sorption on the hydrophobic PNIPAm domain. Overall, with functionalized membranes containing superior surface area-to-volume ratios and enhanced sorption sites, efficient treatment of high-volume contaminated water can be achieved.

## 1. Introduction

Volatile organic compounds (VOCs), such as trichloroethylene (TCE) and tetrachloroethylene (PCE), are a wide category of compounds utilized in various industrial processes, such as paint, degreasing solvent, and metal machinery cleaning [1,2], and have caused various contamination issues in groundwater and other water resources. Due to their high volatility, these compounds also pose indoor air contamination concerns. As previously mentioned, trichloroethylene is a well-known VOC with a Henry’s Law constant of 0.10 mol/(kg·bar) [3] and has become a pollutant of focus in recent years due to its prevalent nature (it does not naturally break down in the environment), widespread use, and emerging health concerns. Upon human exposure, TCE has been shown to be highly carcinogenic as well as damaging one’s immune system, central nervous system, liver, kidneys, and developing fetuses [4,5,6]. Humans are typically exposed to TCE via ingestion of contaminated water or inhalation of generated vapor. TCE reaches human resources through improper waste disposal, industrial site spills, landfill dumping of contaminated products, and subsequent leaching into the surrounding soil/water sources [2]. Due to these aspects of TCE, the need for impactful environmental remediation techniques to capture and degrade such pollutants into less toxic compounds is immense and developing technologies is vital to improving both short-term and long-term health benefits.

There are many methods currently being tested for environmental remediation to combat TCE pollution and reduce human exposure to contaminated water. The capture of TCE has been achieved with several materials, such as activated carbon, biochar, and polymeric materials [7,8,9,10]. In various groundwater treatment sites, TCE is often removed by membrane air-stripping (MAS) in packed columns, followed by vapor adsorption by activated carbon. TCE adsorption, though, is limited by several factors, such as (1) the ability to regenerate the adsorption sites (proven difficult without a costly high-temperature process) [8] and (2) subsequent processing, as the captured TCE is still harmful to humans and the materials still need further treatment.

Alternatively, degrading TCE is becoming an appealing option as the pollutant is being detoxified. Destruction via incineration is an effective method of disposal, but it can be costly with the high temperature needed as well as dangerous with the production of other toxic products [11]. TCE degradation has also been achieved with microorganisms under aerobic conditions [12], but is limited by reaction time (>48 h for 60% degradation). A method with promising efficiency and scalability for volatile pollution degradation is utilizing zero-valent iron (ZVI) and palladium (Pd) nanoparticles, which can dechlorinate polychlorinated contaminants via the reductive pathway [13,14,15]. Briefly, ZVI acts as an electron source that generates hydrogen gas from the surrounding water. This hydrogen gas is utilized by the hydro-dechlorination catalyst (Pd) to produce highly reactive hydrogen radicals that dechlorinate the pollutant. It is important to note that this system is limited by the presence of oxygen in the system, as it can oxidize ZVI and eliminate its ability to generate the hydrogen gas necessary for the catalyst. Compared to free particles in a solution, the immobilization of such Fe/Pd particles into a membrane matrix has been favorable due to high particle loading, high-volume convective flow treatment options, reduction of magnetic particle aggregation, and long-term particle stability with reduced oxidation of ZVI [16].

Our group has reported success in functionalizing optimal membranes for reactive nanoparticle immobilization [17,18,19,20]. Base microfiltration (MF) and ultrafiltration (UF) commercial membranes, such as polyvinylidene fluoride (PVDF), have favorable chemical and mechanical stability and have been commonly used in a variety of water/air treatment applications. The surface and pores of these membranes are functionalized through different modification methods (dip coating, layer-by-layer assembly, surface grafting) with a pH/ionic-responsive polymer, such as poly (acrylic acid) (PAA) or poly (methyl methacrylate) (PMMA) [13,19,21]. This incorporates new properties in the membrane, as these polymers are responsive to the surrounding pH levels and certain functional groups deionize at high pH environments (above the pKa value, which is ~4.5 for PMMA). While the change in ionization can generate electrostatic interactions, which control the effective pore diameter of the membrane and the polymer’s transition between a coil and globule phase, this functionalization’s primary purpose is the immobilization of catalytic metal ions and subsequent reduction to nanosized particles. By controlling the protonation of the COOCH_3_ group of PMMA, Fe can be incorporated into the system via ion exchange. The immobilized iron can then be reduced to ZVI using a reducing agent, such as sodium borohydride, and subsequently doped with Pd for a bimetallic catalytic system [18,20].

The interaction between chlorinated organics and the catalytic sites of these membranes, though, has been proven to be hindered by mass transfer limitations [18]. The diffusion of the contaminant to the active site, as well as the overall catalytic activity, can be increased by utilizing the enhanced hydrophobic interactions of poly (N-isopropylacrylamide) (PNIPAm), a thermoresponsive polymer that can transition from hydrophilic (coil-like polymer structure) to hydrophobic (globule-like structure) properties at its lower critical solution temperature (LCST) of ~32 °C [22]. This response is due to different preferences for hydrogen bond interactions between the polymer functional groups, which are more favorable with water (causing hydrogel swelling) and the polymer itself (causing deswelling via water expulsion) below the LCST and above the LCST, respectively [17].

**Figure 1:**
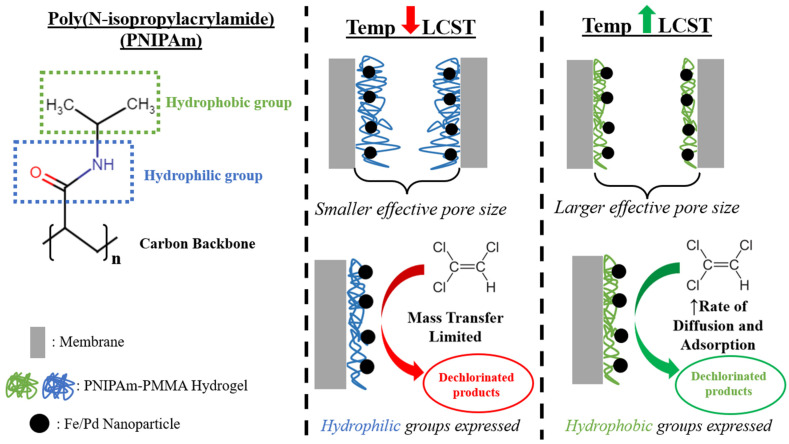
Effect of incorporating PNIPAm into PMMA-Fe/Pd-functionalized membrane systems’ ability to degrade TCE. In this case, TCE is shown as an example of a contaminant, but similar trends with non-chlorinated contaminants, such as methyl orange, can be observed.

In flat sheet membranes, Saad et al. showed that functionalizing the surface and pores of a PMMA-Fe/Pd-PVDF400 membrane with PNIPAm increased the dechlorination rate of polychlorinated biphenyl (PCB-1) by 35% above the LCST [18]. This system also subsequently increased the amount of treatment volume allowed by the functionalized membrane as the effective membrane permeability increased above the LCST due to PNIPAm’s phase transition from coil to globule structure. However, the effects of incorporating PNIPAm into Fe/Pd catalytic membranes for the common pollutants TCE and MO degradation (Figure 1) have not been previously reported in the literature. Furthermore, membrane studies with catalytic and thermoresponsive properties have been limited to flat sheet membranes. Systems with scalability potential (high surface area to volume ratio) and increased flux capabilities (larger water treatment per unit time), such as hollow fiber membranes (HFM) [23], have also not been previously investigated, indicating potential for significant advances in the field of catalytic membrane remediation. HFM systems have shown immense promise for the incorporation of catalytic and magnetic materials, such as osmium NPs in polypropylene (PP) HFMs [24], manganese oxide in PTFE HFMs [25], and magnetic particles impregnated in PP HFMs [26].

Furthermore, TCE is a challenging pollutant for experimentation in the solution phase due to its high volatility and harmful vapor. Azo dyes, such as methyl orange (MO), have been commonly used as model compounds for such pollutants [27,28], as they (1) have a high stability (due to aromaticity) [29], (2) have azo bonds that are easily detected using ultraviolet-visible spectrophotometry (UV-Vis), and (3) are degraded by catalytic nanoparticle systems, thus activity tests can be performed. It is important to note that methyl orange itself is considered a prominent pollutant, with approximately 70,000 tons of dye waste released into water sources every year [30] and certain negative health effects, such as being a contact allergen, an endocrine disruptor, and a possible carcinogen [31,32]. As previously mentioned, the effect of enhancing the degradation of MO with the presence of PNIPAm in a Fe/Pd bimetallic system has not been previously reported.

In this work, the feasibility of integrating PNIPAm into Fe/Pd-PMMA-functionalized hollow fiber membrane systems (with a higher surface area to volume ratio than flat sheet membranes [33]), as well as other flat sheet microfiltration membranes with larger catalyst loading capacities than previously tested, was investigated. PMMA is utilized as the ionic-responsive polymer in this study, it provides scale-up advantages with its considerably lower vapor pressure than PAA [18]. MO has shown usage for initial activity experimentation; thus, MO and TCE degradation by these functionalized materials above and below the LCST of PNIPAm were explored in batch mode. In addition, initial testing was performed to establish that TCE volatilization by hydrophobic HFMs (followed by carbon adsorption) could be an energy-efficient alternative to traditional packed bed stripping. Ultimately, the novelty of this work stems from (1) the functionalization/synthesis of PNIPAm into HFMs, (2) the efficacy of utilizing PNIPAm’s hydrophobic properties (above LCST) in catalytic membranes for previously untested contaminants (such as an azo dye and TCE), and (3) the utilization of hydrophobic HFMs for VOC stripping from groundwater sources.

## 2. Materials and Methods

### 2.1. Materials

Hydrophilized PVDF400 and PVDF650 membranes were provided by Solecta Membranes (Oceanside, CA, USA). The hollow fiber membranes (Tribore, 3M, and Lifestraw) were obtained from START Center Singapore (Cleantech One, Singapore), Quantum Flow Technologies (Charlotte, NC, USA), and Vestergaard (Baltimore, MD, USA), respectively. Sodium borohydride (99%, CAS: 16940-66-2), methyl methacrylate (99%, 80-62-6), and N-isopropylacrylamide (2210-25-5) were obtained from Fisher Scientific (Waltham, MA, USA). Iron(III) chloride hexahydrate (10025-77-1) and iron(II) chloride tetrahydrate (13478-10-9) were obtained from Alfa Aesar (Tewksbury, MA, USA). Sodium chloride, hydrochloric acid, ethanol, sodium hydroxide, and methyl orange (547-58-0) were obtained from VWR (Atlanta, GA, USA). Ammonium persulfate (APS, 98% purity, 7727-54-0) was obtained from Acros Organics (NJ, USA). Potassium tetrachloropalladate(II) was obtained from Strem Chemicals (Newburyport, MA, USA, CAS: 10025-98-6). Trichloroethylene (79-01-6) and N,N′-methylenebisacrylamide (110-26-9) were obtained from Sigma Aldrich (St. Louis, MO, USA). Deionized ultrafiltered (DIUF) water was obtained from a PURELAB Flex 3 filtration system (4–18 ΜΩ). Deoxygenated water was obtained by bubbling nitrogen gas in DIUF water for 30 min.

### 2.2. Hydrogel and Nanoparticle Synthesis

PNIPAm/PMMA hydrogels were created via temperature-initiated free radical polymerization. A polymerization solution was made by adding 13.5 g of NIPAm monomer to 100 mL of DIW. The DIW was bubbled with nitrogen gas for 30 min prior to mixture to ensure minimal oxygen presence. MMA and bisacrylamide cross-linkers were added in a 90 (NIPAm):5:5 molar ratio, respectively, and then 2 mol% of APS was added (relative to NIPAm monomer). The solution was then poured into glass vials (with headspace for hydrogel growth) and left in a vacuum oven for 2 h at 70 °C. Hydrogels were then rinsed with DIW (to remove unreacted monomers) and stored in a cold room (2 °C).

Fe/Pd nanoparticles suspended in solution (without membrane matrix) were synthesized by bubbling 35 mL of DIW with N_2_ gas for 30 min. A total of 15 mL ethanol was added to the solution to obtain a 100 mL solution. A total of 0.284 g of iron(III) chloride hexahydrate was then added to the 50 mL solution (0.035 M) using a mechanical stirrer at 100 rpm. A total of 50 mL of a sodium borohydride solution (0.14 M) was added into the beaker drop wise (5 mL per min) to convert Fe^3+^ to Fe^0^ with cooling packs to prevent temperature rise. This reaction was allowed to occur for 60 min. Then the formed ZVI particles in solution were sealed and sonicated for 5–10 min to allow for the maximum surface area available for Pd interaction. The sonicated solution was then combined with 50 mL of a 5 mol% K_2_PdCl_4_ solution and mixed on an orbital shaker at 300 rpm for 30 min. The formed Fe/Pd particles were subsequently rinsed with ethanol and then stored in an ethanol solution with minimal headspace. Note that mechanical stirring was utilized throughout this process, as the ZVI would stick onto magnetic stirring bars, and, when possible, the solutions were in closed containers to minimize oxidation of the iron particles.

### 2.3. Hydrogel Functionalization of Membranes

For functionalization of membranes with PNIPAm and PMMA, a polymerization solution was created, similar to the free hydrogel polymerization method. Briefly, NIPAm (6 g, 13 wt %), MMA monomer, and bisacrylamide cross-linker were added to 50 mL of DIW (deoxygenated) at a molar ratio of 90:5:5, respectively. After the addition of 1 mol% APS, the membranes were soaked in the polymerization solution for 5–10 min. After soaking, the membranes were then placed between two Teflon plates and heated for 2 h in a vacuum oven at 70 °C. Membranes were then rinsed with DIW (to remove unreacted monomers) and stored in a cold room (2 °C).

The weight gains of the membranes were determined using the dry weights of the membrane before and after polymerization, calculated using the following equation:

(1) $$Weight\;Gain\;\left(\%\right)=100*\frac{m_{final}-m_{initial}}{m_{initial}}$$

where $m_{initial}$ and $m_{final}$ are the dry membrane weights before and after functionalization with polymer. Post-functionalization and DIW rinsing, the membranes were left in a fume hood for 24 h to dry before weighing.

### 2.4. Fe/Pd Nanoparticle Functionalization of Membrane

**Figure 2:**
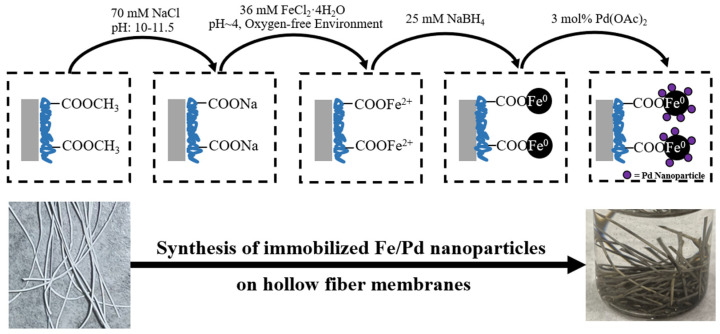
Method summary for ZVI/Pd nanoparticle synthesis and immobilization into a polymer/hydrogel matrix. The depicted HFMs are Lifestraw membranes.

Bimetallic Fe/Pd synthesis and immobilization into the membrane matrix were performed via an ion-exchange method previously reported [18,34], summarized in Figure 2. Briefly, functionalized membranes were soaked in a 70 mM NaCl solution (pH 10–11.5) overnight (12–24 h) to increase the membrane’s ion-exchange affinity with Fe^2+^. The significantly basic pH (above the pKa of PMMA) utilizes the pH response of PMMA, thus allowing the carboxyl functional groups of PMMA to chelate with sodium ions and release H^+^. This exchange can be confirmed by a decrease in pH from the release of hydrogen ions. After this exchange, membranes were soaked in a 36 mM FeCl_2_·4H_2_O solution at 100 rpm for 30 min to ensure ion exchange in the polymer matrix between sodium and iron ions. The membranes were then soaked in a reduction solution (25 mM NaBH_4_ with deoxygenated DIW) for 30 min to reduce the immobilized Fe^2+^ to Fe^0^. Then the membrane was shaken in a 3 mol% Pd(OAc)_2_ solution (10:90 molar ratio of DIW and ethanol to avoid oxidation) at 100 rpm for 30 min for palladium deposition on the iron NPs. The resulting functionalized membranes are washed with ethanol and stored in ethanol with minimal headspace to avoid oxidation of the ZVI NPs.

### 2.5. Responsive Flux Studies of Functionalized Membranes

To examine the permeability of the flat sheet membranes, the membranes were placed in a glass dead-end stirred filtration cell from Millipore. The feed side of the cell was filled with DIUF water and pressurized with ultra-high purity nitrogen gas to create a pressure differential for fluid flow across the membrane. The membrane was compacted at ~1.5 bar until the water flux through the membrane did not change with time. After compaction occurred, the pressure was varied from 0 to 2 bar, and the permeate water volume over specific time intervals was measured at each set pressure. Steady-state flow was achieved before measurements and triplicate samples were taken. Using a linear correlation between the number of liters of water passed per m^2^ of membrane surface per hour versus the pressure difference, the permeability of the tested membrane was determined using the following equation:

(2) $$Membrane\;Permeability=\frac{Q}{A\times \Delta P}$$

where Q (L/hour) is volumetric flowrate, A is membrane area (m^2^), and ΔP is pressure drop across the membrane (bar).

To examine the temperature-responsive nature of the PNIPAm-PMMA-functionalized flat sheet membranes, a heating coil was wrapped around the filtration cell, and a temperature probe was placed inside the cell (as close to the membrane as possible). The permeability of the membrane was measured at different temperature levels. Once the desired temperature was reached, 5 min were allowed to pass before sample measurement, ensuring the thermoresponse and phase transition of PNIPAm.

To examine the pH-responsive nature of the PNIPAm-PMMA-functionalized flat sheet membranes, the membrane permeability was measured with solutions of different pH levels. The pH of the DIUF water was controlled using hydrochloric acid and sodium hydroxide. Five minutes were allowed to pass before sample measurement to ensure the phase transition response of PMMA.

### 2.6. Temperature-Responsive MO and TCE Adsorption/Desorption of Hydrogels

PNIPAm/PMMA hydrogels (1.7 g) were placed in glass vials with ~100 mg/L and ~90 mg/L of MO and TCE, respectively, and shaken at 200 rpm for roughly 24 h (until equilibrium was reached) in an incubated shaker with temperature control. The MO sample was then diluted (1 mL of sample in 50 mL of DIW with 0.1 NaOH for pH adjustment) to reach absorbance values under 1, thus still following the Beer–Lambert Law. pH was also measured and adjusted if necessary to ensure all UV-Vis measurements for MO were taken at the same pH. UV-Vis measurements were conducted using a UV-6300PC (VWR International, Leuven, Belgium). 

Alternatively, the TCE sample was extracted in hexane, 1 μL of the extracted solvent was taken, and it was then diluted 100:1 during GCMS injection. Specifically, for the adsorption study, the incubator was set at 40 °C, which, as previously mentioned, allowed for adsorption to reach equilibrium. After adsorption equilibrium and measurement of concentration (2–3 mL for UV-Vis), the hydrogel was then placed into DIW and shaken again for ~24 h at 23 °C to reach desorption equilibrium. As the hydrogel was in a swollen state below PNIPAm’s LCST, the samples were centrifuged at 2000 rpm for 5 min to separate the hydrogel from the water, and then the separated DIW (with desorbed contaminant) was measured based on the previously mentioned steps.

### 2.7. MO/TCE Degradation via Fe/Pd NPs and Catalytic Functionalized HFMs

For MO degradation using Fe/Pd NPs (free in solution), 0.1 g of bimetallic nanoparticles (ZVI with ~5–10% Pd) was added to 20 mL of a deoxygenated MO solution (3 mg/L), and samples were shaken at 100 rpm below and above the LCST in an incubated shaker. For obtaining a sample at time *t*, the nanoparticles were isolated from the surrounding solution via a heavy-duty magnet (as the Fe/Pd NPs are magnetic), and then 2–3 mL of the sample was removed. MO concentration was analyzed using UV-Vis at a wavelength of 464 nm.

For MO degradation using catalytic functionalized HFMs, 20 mL MO solutions of 5 mg/L were created with deoxygenated water. Roughly 0.15 g of functionalized membranes (cut to 4 cm fiber length) were added to each solution and shaken for 60 min total at 200 rpm. At each timepoint, 2 mL was placed in a cuvette, analyzed via UV-Vis, and then added back into the solution to keep the solution volume consistent across experiments. An incubator shaker was utilized to control the temperature of the samples below and above PNIPAm’s LCST.

For TCE degradation using catalytic functionalized HFMs, 42 mL TCE solutions of ~0.4 mg/L were created with minimal headspace. Roughly 0.44 g of functionalized 3M membranes were added to each solution and shaken for 90 min total at 200 rpm, taking 1 mL samples at the following timepoints: 0 min, 5 min, 15 min, 30 min, 60 min, and 90 min. Septa vial caps were utilized, along with a volumetric needle, for taking samples, ensuring minimal TCE was lost due to volatility. An incubator shaker was utilized to control the temperature of the samples below and above PNIPAm’s LCST. A control without membranes was utilized to normalize TCE concentrations based on lost vapor during sampling. A total of 1 mL of hexane was added to the 1 mL samples taken and shaken at 200 rpm for 30 min for solvent extraction. The 1 mL of hexane (now containing TCE) is then separated from the water for analysis. Triplicate measurements were analyzed using an Agilent (Santa Clara, CA, USA) GCMS system (8890 for GC, 5977C for mass selective detector). Note that the TCE concentration was determined from a calibration curve with known TCE standards (Figure S1). The identification of TCE in experimental samples was confirmed using the gas chromatograph (Figure S2a) and the National Institute of Standards and Technology (NIST) library search with the sample’s mass spectrum (Figure S2b).

### 2.8. Membrane-Air Stripping (MAS)

Membrane-air stripping capabilities for TCE were evaluated using a 3M polypropylene hollow fiber membrane module in a recirculating vacuum membrane distillation apparatus. The hollow fiber module contained approximately 5000 4.9 inch fibers. Initial 200 mL TCE (3.7 mg/L) and DIW mixtures were prepared. One sample was covered as a control (for TCE loss due to volatility), and the other sample was used in the recirculation loop. The solution was pumped through the hollow fiber module at 50 mL/min using a peristaltic pump. Inlet, outlet, and flask temperatures were measured, as well as inlet gauge pressure and shell vacuum pressure. The shell side pressure of the hollow fiber module was reduced using a scroll vacuum pump with a water-chilled Liebig condenser operating at 1–2 °C in line to collect some vapors. The solution was circulated through the hollow fiber module for 30 min, and samples were collected from the recirculated flask, control flask, and condensed liquid.

### 2.9. Gas Chromatography-Mass Spectroscopy (GCMS)

An Agilent GCMS system (8890 for GC, 5977C for mass selective detector) was utilized for the analysis of semi-volatile compounds, such as trichloroethylene. High-purity hexane (CAS: 110-54-3, from Millipore Sigma) was obtained and used as the injection solvent. A high-purity (>98%) TCE standard (CAS: 79-01-6, from Millipore Sigma) was utilized to create external standards for a calibration curve, ranging from 0 (blank control) to 10 mg/L of TCE. Solvent extraction of TCE samples was required prior to GCMS analysis. A total of 10 mL of the sample (solution of DIW, TCE, and subsequent formed products if reaction occurred) was placed into a vial with 10 mL of hexane with minimum headspace and shaken at 200 rpm for 1 h to allow for TCE extraction into the hexane solvent. Control runs were conducted at each experiment to obtain an extraction percent, which was then utilized for normalizing the corresponding resulting data. After allowing the two solvents to separate, a syringe was utilized to extract 2 mL of the hexane solvent (now containing TCE) and placed into 2 mL septa-sealed vials. During analysis, 1 μL of the sample (with 2 sample pumps) is injected into the injection port with a 0.2 μL air gap in the syringe. Three washes with hexane were conducted before and after each sample injection to prevent contamination. The oven temperature was set to 250 °C. A solvent delay of 3.2 min was utilized for observing clear TCE peaks. The limit of detection was found to be under 1 μg/L for TCE.

## 3. Results and Discussion

### 3.1. Membrane Functionalization and Nanoparticle Synthesis

#### 3.1.1. Membranes Characteristics

In this study, several flat sheet and hollow fiber membranes were initially investigated for their functionalization and remediation potential. Variation of different membrane characteristics (structure, hydrophobicity, and pore size) was necessary to investigate the scalability of the polymer and NP functionalization processes. Commercial hydrophilized PVDF400 and PVDF650 membranes were utilized for flat sheet testing with a volumetric water permeability, measured in liters per m^2^ per hour (LMH), found to be 969.4 ± 175.7 and 2557.3 ± 468.3 LMH/bar, respectively (Figure S3). Both membranes contain an asymmetric structure, meaning the pore size varies with membrane thickness (thin PVDF separating layer on an open polyester backing support, highlighted in Figure S4). Commercial hydrophobic PVDF (Tribore-HFM), hydrophobic polypropylene (3M-HFM), and hydrophilized polyether sulfone (Lifestraw-HFM) were utilized for HFM testing. The characteristics of the membranes functionalized in this study are highlighted in Table 1, including material, mean pore size, porosity, and thickness. It is important to note that HFMs will typically have different pore sizes depending on the surface side. The shell side is the outer surface layer of the membrane, while the lumen side is the inner surface (Figure S5).

#### 3.1.2. PNIPAm-PMMA Functionalization of Membranes

PNIPAm is a temperature-responsive polymer that can undergo a conformation change at its LCST (~32 °C). Above the LCST, the isopropyl groups of PNIPAm initially dehydrate, with a subsequent backbone collapse. This leads to the hydrophilic amide groups preferring to hydrogen bond with each other rather than the surrounding water, thus transitioning to a globule-like hydrogel state and releasing previously bound water [37,38]. Quantification of this transition has been conducted previously in our group with swelling studies below and above the LCST with PNIPAm-PMMA hydrogel systems [18]. The purpose of PNIPAm functionalization of the membrane surface and pores is to control membrane pore size (and thus permeability), as well as have the capability to regulate primarily hydrophilic or hydrophobic properties of the membrane system. The purpose of PMMA functionalization, in addition to pH responsiveness to membrane pores and surfaces, is for Fe/Pd NP immobilization into the membrane matrix via ion exchange, which does not allow for the significant release of metals during remediation processes (Wan et al. reported less than 2% leaching in similar systems [39]). This mitigates one of the main concerns with this technology, which is the release of iron and palladium into treated water. Furthermore, pH-responsive polymer functionalization has been known to increase the antifouling properties of the membrane due to the control of surface charge via the pH of the surrounding solution [40,41]. It is important to note that this functionalization method is transferable to other membrane materials. The process does not utilize covalent attachment between the hydrogel and membrane surface; thus, a variety of membrane materials can be utilized. Long-term stability of membrane functionalization with hydrogel can be a concern due to the hydrogel not being chemically attached to the membrane. Many studies, such as one by Chiao et al. [42], have proven that ionic-responsive polymers, such as PAA and PMMA, can be covalently grafted onto polymeric membranes to increase system stability. There are certain trade-offs for implementing this method, though, such as higher functionalization costs and more complex methods, which can decrease the scalability of the resulting systems.

**Figure 3:**
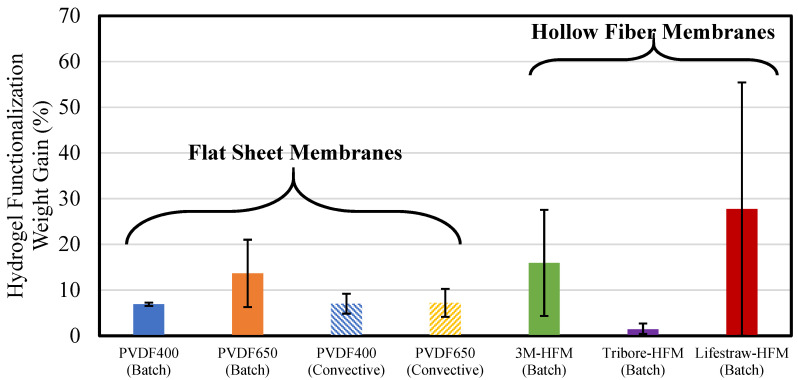
Hydrogel functionalization weight gains of membrane systems. Batch functionalization indicates only the soaking of membrane in polymerization solution, while convective indicates both soaking and convective flow with polymerization solution. Error bars represent the standard deviation of triplicate experiments.

All five membranes in this study were functionalized with PMMA and PNIPAm polymers via a free hydrogel polymerization method. The degree of functionalization was initially quantified by weight gain percent before and after the polymerization process, utilizing Equation (1) (Figure 3). Further confirmation of PNIPAm and PMMA presence was obtained using FTIR (Figure S6 and Supplementary Materials: Section C), where the PNIPAm-PMMA’s -NH_3_ group (~1540 cm^−1^) and -C=O group (~1650 cm^−1^) were identified on the functionalized PNIPAm-PMMA-3M HFM.

With average weight gains above 15%, HFMs displayed a higher affinity (on average) than flat sheet membranes, due to HFMs’ higher specific surface area per gram of membrane [43]. The Lifestraw exhibited the highest average weight gain (27.7%), which can be explained due to the hydrophilization of the polyether sulfone. In this surface/pore coating process, hydrophilic membranes allow for greater coating with the polymerization solution. Alternatively, hydrophobic membranes have low coating affinity. Due to their hydrophobic nature, the Tribore and 3M membranes yielded lower functionalization weights than the hydrophilized Lifestraw, with 1.4% and 15.9%, respectively. There was a significant difference between the two hydrophobic HFMs, though, which indicated that specific surface area and hydrophobicity could not be the sole factors affecting weight gain. Membrane materials (polypropylene and PVDF) could largely affect this process. Overall, these findings indicate that further investigation could be beneficial regarding the effect of membrane variables on the membranes’ hydrogel functionalization capabilities as well as the affinity of the functionalization material (i.e., PMMA) for certain chemical bonds of the membrane material (C-H versus C-F).

#### 3.1.3. Fe/Pd Synthesis and Immobilization into the Membrane System

**Figure 4:**
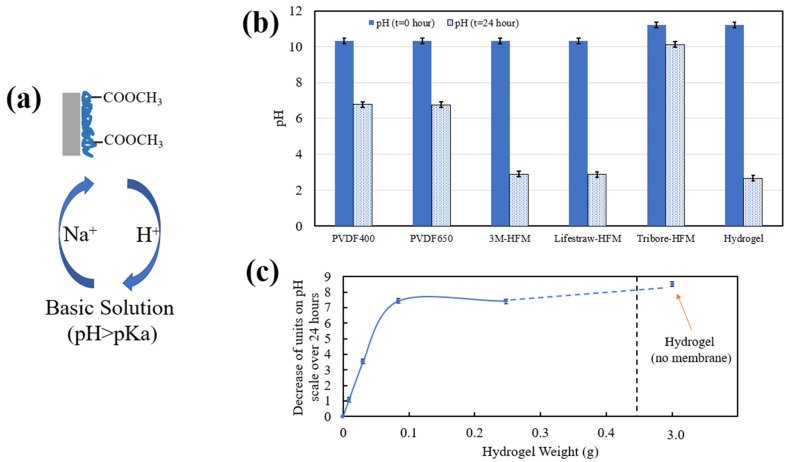
pH-responsive nature of PNIPAm-PMMA hydrogel and functionalized membranes. (**a**) Visual representation of ion exchange for the first step of Fe/Pd immobilization into the hydrogel/membrane domain. (**b**) pH change of solution before and after soaking PNIPAm-PMMA-functionalized membranes for 24 h. A total of 3 g of PNIPAm-PMMA hydrogel was utilized as a reference. The initial pH was set at 10–11. Error bars represent triplicate measurements. (**c**) Correlation of membrane functionalization weight and release of hydrogen ions quantified as decrease of units on the pH scale over 24 h of membrane/hydrogel soaking. Error bars represent triplicate measurements.

After PNIPAm and PMMA functionalization, immobilization, and synthesis of Fe/Pd NPs in the membrane matrix were conducted. As mentioned in the “Materials and Methods” section, the first step involved soaking the membranes in a basic solution (below the pKa of PMMA) with 70 mM NaCl to allow for an ion exchange between hydrogen and sodium (Figure 4a). Due to the release of hydrogen ions, this process decreased the overall pH of the soaking solution (Figure 4b), indicating successful PMMA functionalization, ion exchange, and membrane pH responsiveness. The pH of the 3M and Lifestraw HFMs dropped the most (from roughly 10 to 3) due to their highest average weight gain. Alternatively, the flat sheet membranes with a lower weight gain showed a less significant pH drop (from roughly 10 to 7), and the Tribore, with the lowest weight gain, showed a miniscule pH drop (from 11 to 10). Overall, this pH drop was proportional to the functionalization weight exhibited by each membrane (Figure 4c), up to a solution pH of roughly 2–3, where the released hydrogen ions do not significantly decrease the pH further. This data indicated that, up to a weight gain of roughly 0.1 g of polymer, a linear trend between membrane functionalization weight and pH drop can be developed. It is important to note that error bars for these figures represent triplicate measurements and that unfunctionalized membranes do not produce a pH drop over 24 h.

#### 3.1.4. Functionalized Membrane Characterization via SEM and EDX

After the initial ion exchange step, a second exchange occurred with Fe^2+^ (confirmed with ICP, Supplementary Materials: Section C) and then reduction to ZVI with subsequent immobilization of palladium onto the NPs. After functionalization studies, PVDF650 was selected as the flat sheet membrane of focus in this study due to its larger pore size and permeability than PVDF400 (previously mentioned in Figure S3), which can result in greater NP loading and daily water treatment volume. The 3M system was selected as the HFM of focus in this study due to its high functionalization weight and release of hydrogen ions, confirming significant PMMA presence, as well as its hydrophobicity for TCE stripping potential via MAS. While the Lifestraw HFM system had a greater weight gain than that of the 3M, the 3M fibers are held together by perpendicular microfibers (Figure S7), which increases the structural integrity of the fibrous system and is more appealing for commercial and scalable modules. Additionally, the Lifestraw functionalization (~20 nm surface pore size post-functionalization) showed a much larger inconsistency during functionalization (large error bars) than that of the 3M, making the latter a more consistent functionalization material. It is important to note that, to the group’s knowledge, this is the first reported case of HFM functionalization with both catalytic NPs and the thermoresponsive polymer PNIPAm, incorporating both adsorptive and degradative properties for highly efficient water remediation in a hollow fiber system.

**Figure 5:**
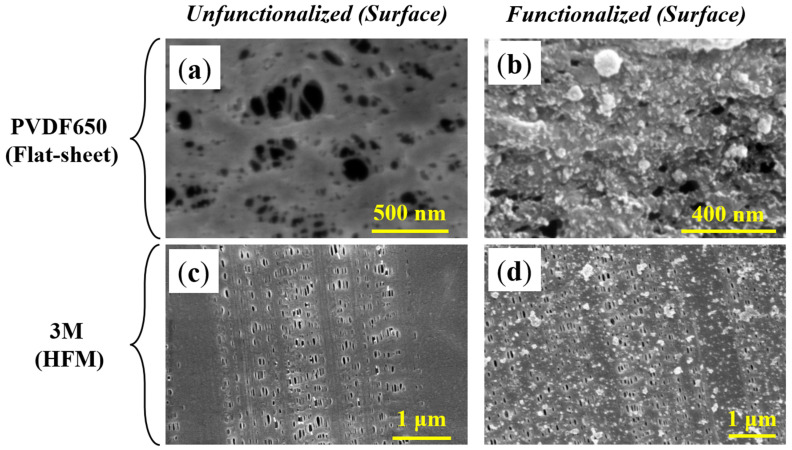
SEM analysis of flat sheet and hollow fiber membranes. Imaging of (**a**) an unfunctionalized PVDF650 surface, (**b**) a PVDF650 surface functionalized with PNIPAm, PMMA, and Fe/Pd NPs, (**c**) an unfunctionalized 3M HFM surface, and (**d**) a 3M HFM surface functionalized with PNIPAm, PMMA, and Fe/Pd NPs.

The Fe/Pd-PNIPAm-PMMA-functionalized membrane systems of focus were characterized using SEM imaging to investigate surface/pore morphology as well as Fe/Pd NP presence (Figure 5), which were found to have an average particle diameter of 33.6 nm and 34.8 nm for PVDF650 and 3M HFM, respectively (Figure S8). Note that the NPs observed on the surface were confirmed to be Fe/Pd nanoparticles via EDX analysis, with respective peaks for both Fe and Pd.

The surface imaging of the PVDF650 membrane showed significant pore functionalization, as the surface pores are less frequent and smaller in diameter post-functionalization (Figure 5a,b). This phenomenon was also confirmed experimentally, as the PNIPAm, PMMA, and Fe/Pd functionalization of the PVDF650 membrane decreased the water permeability from around 2500 LMH/bar to about 824 LMH/bar, indicating significant functionalization of the membrane pores. Due to the complexity of HFM modules that can support water filtration pressures, flux data for HFMs before and after functionalization, as well as temperature/pH response, was not investigated, which is a limitation of this current work. A significant reduction of membrane permeability is not expected for the 3M HFM, though, as the pores do not show significant interference post-functionalization (Figure 5c,d). This difference can be explained by the hydrophobic nature of the 3M fibers (compared to the hydrophilic PVDF650). After investigation of the 3M membrane pores (cross-section) using SEM and ion milling, no NPs were found throughout the pores (Figure S9), which indicates that the majority of functionalization occurred on the shell surface of the 3M membrane and only minimally, if any, in the pores and lumen side. Due to this observation, the recommended direction for convective filtration with the catalytic membranes in this study is shell-to-lumen flow. It is important to note, though, that other sections of HFMs can be functionalized as well, such as lumen-side functionalization with NPs in a packed-bed-style format [44]. Additionally, with ImageJ, the average diameter of Fe/Pd NPs was found to be 33.6 nm and 34.8 nm, respectively, for the PVDF650 and 3M membranes (Figure S9), which is similar to previous membrane-bound Fe/Pd studies [18]. Note that the previous numbers are mean values, and larger particles (diameter: 100–200 nm) were also observed.

Free Fe/Pd NPs (in solution) were also synthesized for initial activity tests. Briefly, the initial oxidized iron solution was reduced via sodium borohydride, where ZVI NP formation occurred (Figure S10). An initial check of this step was conducted visually with a color change from orange/brown to black, which corresponds with Fe^3+^ and ZVI, respectively, in the literature. Further confirmation was obtained with magnetic testing. Unlike Fe^3+^, the formed ZVI nanoparticles displayed paramagnetic properties [45], as shown in Figure S10. After ZVI formation, a palladium acetate solution (3 mol%) was then added for palladium reduction (from ZVI) and subsequent deposition onto the NP surface. It is important to note that our group has previously reported X-ray diffraction (XRD) characterization of ZVI NPs prepared utilizing the same synthesis methodology. This analysis confirmed the formation of zero-valent NP via the peak for body-centered cubic (BCC) Fe^0^ (Figure S11) [46].

The resulting Fe/Pd particles were characterized using TEM and EELS (Figure S12), where the zero-valent iron core was observed with a thin oxygen layer surrounding it. EELS analysis confirmed bimetallic NP formation (not separate Fe and Pd NPs), as a clear Fe core region with Pd coating was observed, which agrees with TEM/EELS analysis in the literature [47]. It is important to note that the formation of Pd NPs had to occur on the ZVI NP surface (thus forming a bimetallic system), as the Pd must be reduced by the ZVI for Pd NP formation (Fe^0^ + Pd^2+^ → Fe^3+^ + Pd^0^) with subsequent deposit on the iron NP surface [47].

Using EELS, the weight percent composition of a single NP was found to be 12.11%, 80.11%, and 7.77% for oxygen, iron, and palladium, respectively (Table S1), further confirming bimetallic NP synthesis. Additionally, the synthesized particles had an average size of 36 nm (Figure S13). Note that NPs formed in membrane systems have a lower average size than those formed unbound in solution, due to the prevention of NP agglomeration (from PMMA charge groups).

### 3.2. Temperature and pH Response of Functionalized Materials

In this study, unbound hydrogels were synthesized using the polymerization solution, consisting of MMA and bisacrylamide cross-linker in a 90 (NIPAm):5:5 molar ratio, respectively, with an overall APS molar percentage of 8%. It is important to note that the degree to which the hydrogel can swell and respond to external stimuli is dependent on the degree of polymer cross-linking and monomer concentration, which can be controlled by the molar ratio of the cross-linker. The swelling capacity of the hydrogels can be described as:

(3) $$S=\frac{d_{swollen}}{d_{unswollen}}$$

where S is the swelling capacity, described as the ratio of the apparent hydrogel diameter in the swollen state (below LCST) to that in the unswollen state (above LCST). Swelling capacity was found to be approximately 10.3 and 3.4 for 3 mol% and 10 mol% crosslinkers in prior studies [18], respectively, which indicates the inverse relationship between swelling capacity and crosslinking concentration in the polymerization solution. This also indicates that, as the degree of crosslinking increases, the responsive nature of the hydrogel subsequently decreases, as the hydrogel does not have the flexibility to swell/shrink.

**Figure 6:**
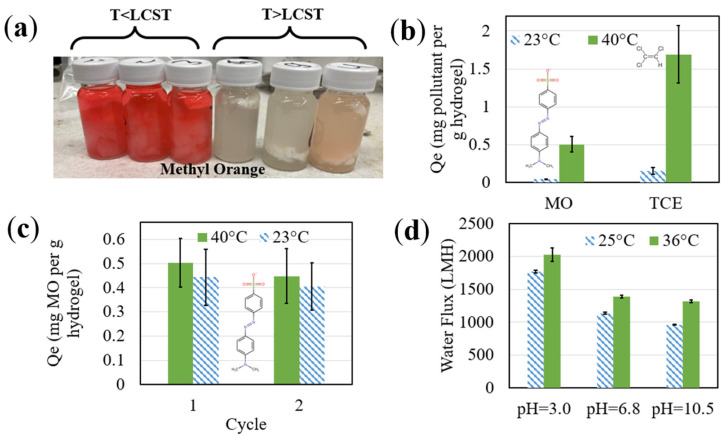
Methyl orange adsorption interactions with PNIPAm/PMMA hydrogel and flux studies of PNIPAm/PMMA-functionalized flat sheet membranes. (**a**) MO (indicated by a red color) solution with PNIPAm-PMMA hydrogel below and above the LCST of PNIPAm. Adsorption of (**b**) MO and TCE onto PNIPAm-PMMA hydrogels below and above the LCST at a pH of 7–8, as well as (**c**) two temperature swing cycles with desorption below the LCST at a pH of 7–8. Note that the 40 °C of cycle 1 corresponds to the same hydrogels as the 40 °C samples from (**b**). Green bars represent experimentation at 40 °C, and blue-lined bars represent experimentation at 23 °C. The initial concentrations of MO and TCE were ~100 mg/L and 93 mg/L, respectively, and the weight of the hydrogels utilized was approximately 1.7 g. Error bars represent triplicate samples, except for TCE data, which represents duplicates. (**d**) Volumetric water flux changes of a PNIPAm-PMMA-PVDF650 flat sheet membrane in response to variation in temperature and pH. Green bars represent experimentation at 36 °C, and blue-lined bars represent experimentation at 25 °C. Note that these flux values are corrected for changes in viscosity with respect to changes in temperature. Water flux is measured at 1.4 bar with an unfunctionalized membrane flux of 3580 LMH. Error bars represent triplicate samples.

Temperature-responsive adsorption of TCE onto hydrogels has been reported by Xiao et al. with 80:10:10 NIPAm:AA:poly (ethylene glycol) 600 dimethacrylate molar ratios, resulting in almost twice as much adsorption of TCE onto PNIPAm-poly (acrylic acid) hydrogels above than below the LCST [48]. The thermoresponsive adsorption/desorption properties of hydrogels that substitute poly (acrylic acid) with PMMA have not been previously investigated with MO or TCE compounds. In this study, free PNIPAm-PMMA hydrogels were utilized for initial responsive experimentation before membrane testing. Equilibrium adsorption isotherm studies were conducted with hydrogel samples in batch solutions of MO and TCE to determine the equilibrium adsorption (Q_e_) of contaminants onto the responsive hydrogel below and above the LCST of PNIPAM. Note that the molar concentrations of TCE and MO are not equal; thus, comparisons were simply made for the investigation of responsive LCST behavior. Initially, Q_e_ was determined to be 0.044 ± 0.003 mg of MO per gram of hydrogel sample at 23 °C. Above the LCST, though, at 40 °C, the value for Q_e_ dramatically increased to 0.504 ± 0.101 mg/g, indicating a 11.5× increase in adsorptive capabilities (Figure 6a,b). For TCE, Q_e_ was determined to be 0.156 ± 0.046 mg of TCE per gram of hydrogel sample at 23 °C. Above the LCST (40 °C), the value for Q_e_ increased to 1.692 ± 0.383 mg/g, indicating a 10.8× increase in adsorptive capabilities (Figure 6b). This increase can be attributed to the dehydration of the PNIPAm chains, which resulted in increased exhibition of hydrophobic properties/groups and greater interaction between the PNIPAm and the hydrophobic sections of the pollutants. It is important to note that PMMA itself is temperature-responsive [49], but it is assumed to have negligible effects on the system due to the low molar ratio of MMA to NIPAm. Despite this, drastically larger Q_e_ were observed at higher temperatures, indicating that hydrogel hydrophilicity was primarily controlled by the responsive PNIPAm chains and that their dehydrating isopropyl groups dominated the more hydrophilic PMMA groups. Additionally, note that the molar concentrations of TCE and MO are not equal; thus, comparisons were simply made to prove responsive LCST behavior.

Additionally, the same hydrogels (utilized in 40 °C MO adsorption in Figure 6b) were returned to temperatures below the LCST over a total of two temperature swing cycles and shaken for ~24 h until equilibrium. In these cycles, minimal desorption was observed, with less than 20% of adsorbed MO released back into the surrounding solution (Figure 6c). The low desorption could be attributed to the strong hydrophobic interactions remaining, the entanglement of the pollutant in the polymer chains, and the bulky size of MO (327.33 g/mol) hindering the mass transfer, compared to the smaller contaminant PCB-1 (188.7 g/mol) that had a higher desorption capacity in previous studies [17,18]. Overall, the efficacy of implementing PNIPAm for increased interaction between the active site and the contaminants MO and TCE above the LCST was shown, as well as the thermoresponsive nature of the hydrogel itself.

The pH responsiveness of the functionalized membrane samples was shown prior to the Fe/Pd immobilization process with the release of hydrogen ions and subsequent pH drop in a solution with a pH > pKa value of PMMA. This response validated the presence of carboxylic groups in the hydrogel domain of the membrane system. Temperature responsiveness, as well as additional pH responsiveness, was shown in the flat sheet PVDF650 membrane with water flux changes at different temperatures and pH levels (Figure 6d). During the transition from below to above the LCST of PNIPAm, the permeability of the functionalized PVDF650 membrane increased by approximately 14%, 22%, and 37% for pH levels of 3.0, 6.8, and 10.5, respectively. These changes indicate the thermoresponsive nature of membranes with functionalization. It is important to note that these flux values were obtained after undertaking viscosity corrections with respect to temperature; thus, flux changes below and above the LCST were solely due to the swelling/shrinking of the PNIPAm chains, respectively. Furthermore, below the pKa of PMMA, the PMMA chains are in a collapsed state, which restricts the responsive nature of the PNIPAm chains. This is seen in the sample at a pH of 3.0 (below the pKa), which had the lowest increase in flux transitioning above the LCST. Overall, these differences indicate the successful pH-responsiveness of the membrane after functionalization.

### 3.3. Pollutant Degradation with Responsive Catalytic Membranes

#### 3.3.1. Steps of the Reductive Degradation Pathway with Corresponding Models

The degradation of TCE with ZVI systems is typically modeled using pseudo-zero-order or first-order reaction rate kinetics with high and low concentrations of TCE, respectively [50]. In this study, the concentrations of pollutants are relatively low, so the following pseudo-first-order (PFO) reaction rate relationship was utilized for degradation modeling:

(4) $$-\frac{dC}{dt}=C\times k_{obs}=C\times (k_{sa}\rho_{m}a_{s})$$

where $k_{obs}$ is the observed reaction rate constant (1/min), $\rho_{m}$ is the iron loading density (g/L), $a_{s}$ is the nanoparticle surface area to mass ratio (m^2^/g), and $k_{sa}$ is the bimetallic nanoparticle surface area-normalized reaction rate constant (L/m^2^/h). It is important to note that PFO rates have certain limitations, as several assumptions are made: (1) negligible mass transfer resistance between the liquid and solid boundary layer, (2) irreversible degradation of TCE, DCE, and other intermediates, (3) TCE adsorbed onto the NP surface dechlorinates before product desorption can occur, (4) all reactions are isothermal, (5) no competitive effects between pollutant and product species, and (6) products in gas form (i.e., ethylene) are present only in headspace and not in solution (due to low aqueous solubility) [51].

**Figure 7:**
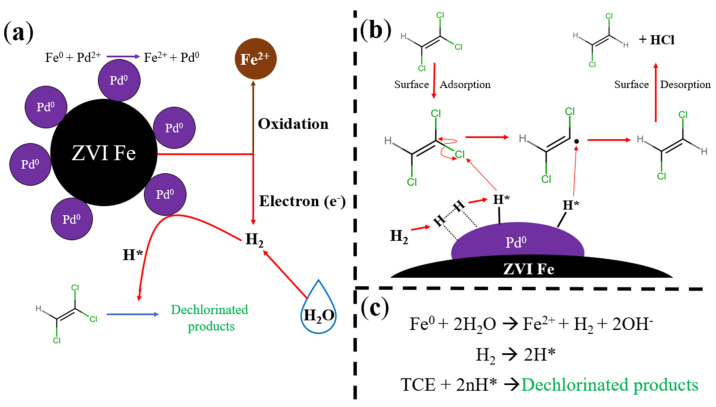
Schematic detailing degradation of TCE via Fe/Pd NPs. This schematic contains (**a**) an overview of the processes with a detailed explanation of hydrogen gas generation from iron corrosion, (**b**) the multi-step process of the TCE contaminant, including adsorption, degradation, and desorption, and (**c**) simplified stoichiometric reaction steps for the overall process. The “*” symbol represents a radical.

To create a more accurate mode, the specific phases of this pathway must be considered. The degradation of TCE via Fe/Pd NPs occurs through several steps, summarized in Figure 7a. Initially, the ZVI is oxidated (converting to Fe^2+^ or Fe^3+^), which releases electrons. Through a hydrogen evolution reaction, these electrons produce hydrogen gas from the surrounding H_2_O in addition to hydroxide (OH^−^). H_2_ gas is then adsorbed onto the surface of the Pd^0^, along with the contaminant TCE, where hydrogen radicals are produced. These hydrogen radicals then hydrodechlorinate the TCE contaminants to DCE, ethylene, and eventually ethane ($C_{2}H_{6}$).

Several mathematical models have been used for the degradation of TCE via Fe/Pd NPs in batch mode. Summarized in Figure 7b in simplistic terms, there are three main steps to this process that the model can include [51]:

(1) Mass transfer of TCE ($C_{2}HCl_{3}$) from the bulk solution to the surface of the catalyst site and subsequent surface adsorption, forming a di-σ-bonded species from the intermediate (π-bonded):

$$C_{2}HCl_{3}+S\;\left(active\;site\right)\;\;\underset{\;k_{1a}}{\overset{k_{-1a}}{⇋}}\;\;C_{2}HCl_{3}-S$$

$$C_{2}HCl_{3}-S+S\;\to \;C_{2}HCl_{3}*-S_{2};$$

(2) Hydrodechlorination of TCE on the active sites of the NPs with DCE as a product and eventually ethane:

$$\frac{5}{2}Fe^{0}+5H_{2}O+5Pd\to \frac{5}{2}Fe^{2+}+5OH^{-}+5Pd-H$$

$$\frac{3}{2}Fe^{0}\to \frac{3}{2}Fe^{2+}+3e^{-}$$

$$C_{2}HCl_{3}*-S_{2}+5Pd-H+3e^{-}\;\to \;C_{2}H_{6}*-S_{2}+5Pd+3Cl^{-};$$

(3) Generated products desorb from the catalyst surface and transport into the surrounding bulk solution:

$$C_{2}H_{6}*-S_{2}\to \;C_{2}H_{6}+S_{2}.$$

In the second step, note that the hydrogen generation originates from the oxidation of the ZVI but is a short-lived product and is not included as an individual step. Overall, this pathway, along with the basic overall reaction steps in Figure 7c, is a simplified version of the complete process and can be affected by several other factors. For example, the degradation of TCE produces several compounds that can compete for active sites on the NP surface if their concentration is high enough [50].

Alternatively, for methyl orange, Fe/Pd NPs facilitate the cleavage of the azo bond (-N=N-) to -NH groups [52]. This azo bond has a significant absorption peak around 464 nm; thus, degradation and NP activity can be easily quantified by analyzing the reduction of absorption at this peak using UV-Vis (calibration curve available as Figure S14). It is important to note that the desorption step of MO is slower than that of TCE, meaning that significant adsorption of MO onto the NP surface occurs [53], which is a limitation of utilizing MO as a model compound.

#### 3.3.2. Effect of Temperature on MO Degradation via Fe/Pd Nanoparticles

Bulk diffusion plays a significant role in the degradation process of MO and TCE via Fe/Pd immobilized in membrane systems and can be modeled using the Wilke–Chang model or the Stokes–Einstein equation. The Stokes–Einstein equation is described as follows:

(5) $$D_{s}=\frac{k_{B}T}{6\pi \mu r_{part}}$$

where $D_{s}$ is the bulk diffusion coefficient, $k_{B}$ is the Boltzmann’s constant, T is the temperature (K), $r_{part}$ is the solute molecular radius, and μ is the dynamic viscosity of the solvent. Utilizing the Stokes–Einstein equation, the bulk diffusion coefficients from 5 to 50 °C have been calculated for MO, TCE, and polychlorinated biphenyl (PCB-1, used in prior studies [18]) in a water solvent in Figure S15. In summary, the bulk diffusion coefficients of MO and TCE at 25 °C were found to be 5.5 × 10^−11^ and 9.1 × 10^−10^ m^2^/s, respectively, while at 40 °C, they were found to be 7.8 × 10^−11^ and 1.3 × 10^−9^ m^2^/s, respectively. This proves that diffusion itself will increase with system temperature elevation. Additionally, it is important to note that the larger diffusion coefficient of TCE compared to MO can be explained by TCE’s smaller molecular size, which decreases resistance to mass transfer.

**Figure 8:**
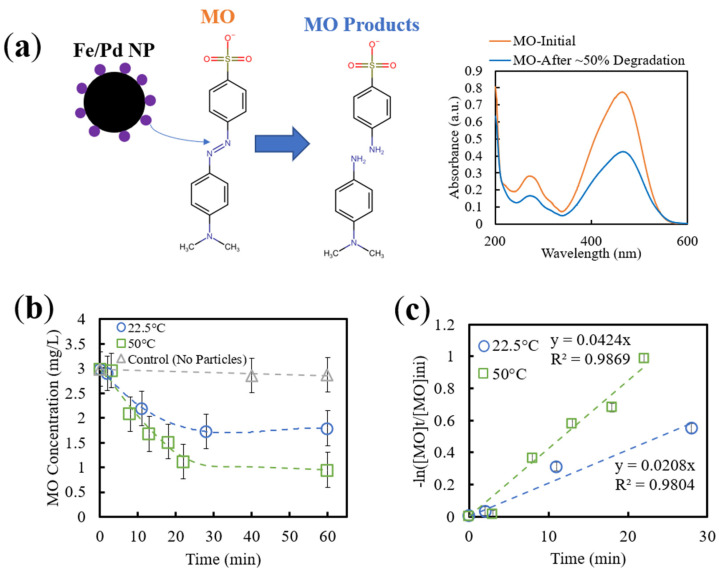
MO degradation with Fe/Pd NPs in a solution. (**a**) Schematic of interaction between Fe/Pd NP and MO, as well as an example of UV-Vis absorbance spectrum before and after degradation from NPs (initial concentration: ~10 mg/L MO). (**b**) MO degradation of Fe/Pd NPs at different temperatures with respect to reaction time. A total of 0.1 g Fe (~10% Pd) in 20 mL vials was used with a MO concentration of 3 mg/L and a pH of 6.8. Samples were shaken at 100 rpm. Error bars indicate triplicate measurements. (**c**) Pseudo-first-order model of MO degradation via Fe/Pd NPs in solution at two different temperatures. Error bars indicate triplicate measurements.

The activity of Fe/Pd NPs free in solution at both 22.5 °C and 50 °C was initially tested with MO to verify the activity of the nanoparticles, the validity of MO as a model pollutant, the fit of a PFO model, and investigate particle fouling with the pollutant. For pollutant sampling, the nanoparticles (with paramagnetic properties) were briefly separated from the surrounding solution via a strong magnet. MO concentration was then analyzed using UV-Vis at a wavelength of 464 nm, representing the N=N bond that was reduced upon the Fe/Pd reaction (Figure 8a).

As expected, more rapid degradation of MO occurred at 50 °C than at 22.5 °C (Figure 8b), which can be explained by the increased MO diffusivity at the higher temperature (Stokes–Einstein equation). Oxidation of ZVI as well as NP fouling via adsorbed MO (and subsequent products after reduction) can be observed as the degradation process begins to deviate from first-order kinetics after approximately 25 min of reaction time. Low desorption rates (7%) have been reported by Arshadi et al. with MO and metal nanoparticles, which can be attributed to the formation of a strong metal-dye complex [54]. Additionally, there is a small initial delay in degradation activity, which can be explained by the transport limitations of MO through the oxygen layer surrounding the NPs as well as the H_2_O interacting with the Fe core and subsequent hydrogen generation.

The experimental data was fitted to a pseudo-first-order model to quantify reactivity and obtain reaction rate kinetics (Figure 8c). Utilizing data until degradation stopped (20–30 min of reaction time), the observed reaction rate (k_obs_) was determined from the slope of the linear fit to be 0.0208 and 0.0424 min^−1^ for 22.5 °C and 50 °C, respectively (Table 2). A more accurate representation of reaction rate can be obtained by calculating the surface area-normalized reaction rate constants (k_sa_), as it normalizes the constants with respect to NP loading and surface area (average obtained from TEM imaging). It is important to note that the values for loading and surface area of NPs can vary significantly depending on membrane weight gain after hydrogel functionalization (available sites of Fe ion exchange and immobilization) and formed NP size (surface area ratio compared to mass of NP). Using Equation (4), k_sa_ was calculated to be 0.0118 and 0.0240 L/m^2^/hour for 22.5 °C and 50 °C, respectively, indicating a ~two-fold increase in reaction rate constants from this temperature increase. To eliminate the effect of temperature on the model values, these constants can be normalized with respect to temperature using the Arrhenius equation:

(6) $$\ln\frac{k_{2}}{k_{1}}=\frac{-E_{a}}{R}\left(\frac{1}{T_{2}}-\frac{1}{T_{1}}\right)$$

where R is the gas constant, T is the temperature (K), and $E_{a}$ is the activation energy (kJ/mol). After normalization with respect to 22.0 °C, the k_sa_ values were roughly equal (Table 2), indicating that there were no other forces affecting the reactivity at different temperatures besides changes in contaminant diffusion. Overall, MO was confirmed to be an appropriate model for Fe/Pd NP systems, and the effect of temperature on diffusivity was investigated with appropriate kinetic models, yet a possible limitation of utilizing MO as a model compound was found (low MO desorption from the NP surface).

#### 3.3.3. Thermo-Responsive Degradation of MO via Catalytic Membranes

The Stokes–Einstein model is appropriate for batch NP experiments, but with catalytic NPs imbedded into a membrane/hydrogel matrix, the diffusion rate will be significantly lower due to the crosslinked hydrogel matrix and membrane material. Yang et al. developed a model that can be fitted to the obtained data for contaminant diffusion in the presence of a catalytic membrane [56]:

(7) $$\frac{C_{TCE}}{C_{TCE,initial}}=\left(\frac{DH}{l}\right)\left(\frac{A}{V}\right)\left(t-\frac{l^{2}}{6D}\right)$$

where D is the diffusion coefficient (m^2^/s), H is the partition coefficient, A is the membrane area (m^2^), V is the permeate solution volume (m^3^), and t is the time (s). Using Equation (7), the diffusion coefficient can be derived from the slope and intercept. It is important to note that, as previously mentioned, several literature sources have found this catalytic process to be diffusion-limited [39,57,58], which is the motivation for PNIPAm incorporation. Significant differences in diffusion coefficients have been reported for PNIPAm-functionalized membranes in the literature, as 6.6 × 10^−11^ and 8.7 × 10^−11^ m^2^/s at 25 and 35 °C, respectively, for PCB-1 [18], showing the validity of Yang et al.’s model and the PNIPAm functionalization.

Similar to diffusion modeling, though, the previous equations are not adapted for responsive membranes. The residence time (Equation (8)) within the membrane varies depending on the temperature and permeate flow rates (which are pressure-dependent) of an Fe/Pd-PNIPAm-membrane as the void fraction (membrane volume multiplied by surface porosity) changes above and below the LCST (due to polymer swelling/shrinking). Wan et al. derived a model to determine the pseudo-first-order reaction constants of such membranes using the following two equations [46].

(8) $$\tau =V_{void}/(J_{w}A)$$

(9) $${\bar{C}}_{final}={\bar{C}}_{initial}\frac{\tau^{2}}{4}\left[k_{obs}{}^{2}\left(\int_{k_{obs}\tau /2}^{\infty }\frac{e^{-t}}{t}dt\right)+e^{-k_{obs}\tau /2}\left(\frac{4}{\tau^{2}}-\frac{2k_{obs}}{\tau }\right)\right]$$

where $V_{void}$ is the membrane void fraction, ${\bar{C}}_{final}$ is the final contaminant concentration, ${\bar{C}}_{initial}$ is the initial contaminant concentration, and τ is the residence time (s).

It is important to note that these models are not without their limitations. Foo et al. present a comprehensive review of different adsorption isotherm systems [59]. Langmuir and Freundlich adsorption isotherms are typically used for such TCE adsorption reactions, with assumptions regarding monolayer vs. double layer adsorption. Furthermore, as previously mentioned, convective flow for HFMs is a current limitation of this work, but previously shown models could theoretically be applied to HFM flow to compare diffusion coefficient changes across the LCST.

The temperature-responsive presence of PNIPAm on the functionalized HFMs containing Fe/Pd NPs was tested with MO in batch mode. The membranes were cut to 4 cm fiber length and shaken for 60 min total in a ~5 mg/L MO solution. An incubator shaker was utilized to control the temperature of the samples below and above PNIPAm’s LCST. Note that the tested membranes had a NP loading of 5.9 mg iron per g of membrane, determined from ICP, with approximately 3 mol% of Pd.

**Figure 9:**
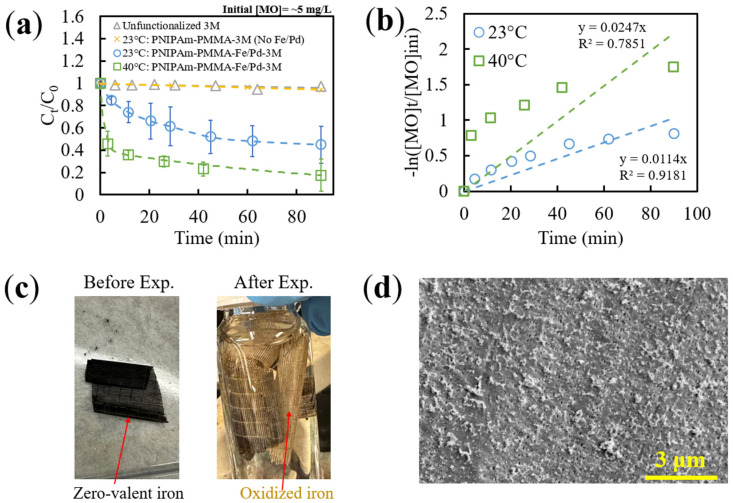
Degradation of MO via PNIPAm-PMMA-functionalized 3M HFMs with 5.9 mg iron per g of membrane and approximately 3 mol% of Pd. (**a**) MO degradation by PNIPAm-PMMA-functionalized 3M HFMs below and above the LCST of PNIPAm in batch mode. Concentration values were normalized based on membrane weight (150–170 mg). Error bars represent triplicate samples. Samples were shaken at 200 rpm, and the solution pH was roughly 6.5. Non-degraded MO was analyzed at a wavelength of 464 nm using UV-Vis. (**b**) Pseudo-first-order fit of MO degradation data by functionalized HFMs. Error bars represent triplicate samples. (**c**) Image of 3M membrane before and after experimentation, indicating significant oxidation of iron (from black to brown color). (**d**) SEM image of functionalized membrane after 90 min of MO degradation.

Similar to free NPs, a more significant MO degradation versus time was observed at a higher temperature of 40 °C (Figure 9a). Note that control absorbance values (from shaking HFM without MO presence, ranging from 0.01 to 0.1) were subtracted from measured values to ensure the change in absorbance was due to MO degradation and not the release of unbound NPs. When fitted to a PFO model, though, a significant deviation occurred (Figure 9b) from a linear fit. This is most likely due to the low desorption rates of MO from the catalytic NPs (as previously mentioned), lower mass transfer rates overall through membrane systems (as opposed to free catalysts in solution), as well as the oxidation of ZVI throughout the duration of experimentation (Figure 9c). This could be an indication of a limitation of the pseudo-models for describing complicated reaction processes. Furthermore, functionalized membranes were characterized with SEM after 90 min of MO degradation, showing significant surface fouling and some loss of iron NP structure (Figure 9d), indicating limitations for long-term MO treatment.

It is important to note that oxidation of the ZVI NPs is a current limitation of this technology, as conversion from ZVI (black-colored membrane) to Fe^2+^ (brown-colored membrane) depletes the electron-donating source. This can lead to a loss of long-term activity, leading to the need for regeneration of the ZVI or the introduction of dilute hydrogen gas. Even though NPs in this study are immobilized in a hydrogel matrix, thus offering some protection from oxidation, the color change in Figure 9c does indicate a certain degree of oxidation. For similar membrane systems, our group has reported less than 20% Fe/Pd initial activity after four degradation cycles over a 3 month period in water [16]. One promising method for overcoming this limitation is the reduction of Fe^2+^ via green tea extract instead of sodium borohydride. This method has been shown to maintain at least 85% of Fe/Pd initial activity in membranes after 3 months of periodic activity testing [16] and could be implemented in future HFM studies.

In this work, an initial Fe/Pd stability test was performed by storing the NPs in DIW with different dissolved oxygen (DO) concentrations (~0 ppm and 5.5 ppm). After 24 h of storage, the samples were analyzed using TEM and EELS (Figure S16). The sample with minimal DO levels showed a prominent iron core with a small oxidative shell surrounding it, indicating no noticeable difference from the initial sample (Figure S12). Alternatively, the 5.5 ppm DO sample showed loss of the iron core with significant NP oxidation as well as the formation of iron oxide sheets, thus displaying a leading example of the long-term oxidative limitations of these systems.

Furthermore, the MO concentration did not decrease for both controls (unfunctionalized HFM and PNIPAm-PMMA-3M without NPs). This indicated that the decrease in MO during experimentation with NP-functionalized membranes can be primarily attributed to catalytic degradation and not adsorption onto the membrane system. Additionally, the initial delay observed in the free NP samples was not apparent in this system, which could be due to the PNIPAm in the system overcoming the previously mentioned mass transfer limitations. Overall, though, it is important to note that, to the group’s knowledge, this is one of the first reported demonstrations of enhancing MO degradation with the presence of PNIPAm in a Fe/Pd bimetallic system. These findings bring significant advances to the remediation field by increasing the range of effective contaminants that these thermoresponsive membrane systems can treat in groundwater sources.

For direct comparison with free NPs as well as with TCE degradation, approximate observed reaction rate constants were obtained from the slope of the linear PFO model fit for these functionalized membranes. When correcting the observed constants with respect to temperature, surface-area-normalized constants were found to be 0.6247 and 0.8147 LMH for 23 °C and 40 °C, respectively (Table 3), which are comparable to previous studies with similar catalytic HFM systems [60]. This 30% increase in reaction rate constants can be attributed to the PNIPAm’s phase transition above LCST (expressing hydrophobic domains), leading to lower mass transfer limitations during remediation. It is important to note that, in comparison to the free NPs, the membrane-bound system had significantly higher k_sa_ values (after particle loading normalization), which further indicates the benefit of the hydrogel domain incorporation and subsequent increased adsorptive properties for degradation efficiency. Overall, the efficacy of utilizing PNIPAm’s phase transition to increase the degradation capabilities of catalytic HFM systems was validated, showing promising advances for scalable environmental remediation devices.

#### 3.3.4. Thermoresponsive TCE Degradation via Catalytic Membranes

After initial testing with the model compound MO, batch degradation of TCE below and above PNIPAm’s LCST was conducted with PNIPAm-PMMA-functionalized HFMs containing Fe/Pd NPs (in vials with minimal to no headspace). Septa vial caps were utilized, along with a volumetric needle, for taking samples, ensuring minimal TCE was lost due to volatility. Additionally, an incubator shaker was utilized to control the temperature of the samples below and above PNIPAm’s LCST. A control without membranes was also utilized to normalize TCE concentrations based on maximum lost vapor during sampling.

**Figure 10:**
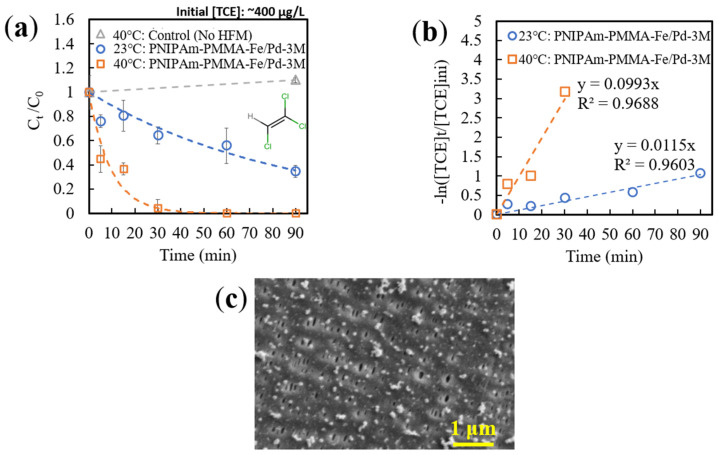
TCE degradation with functionalized membrane systems with 5.9 mg iron per g of membrane and approximately 3 mol% of Pd. (**a**) TCE degradation by PNIPAm-PMMA-functionalized 3M HFMs below and above the LCST of PNIPAm in batch mode. Each vial contained roughly 4.3–4.6 g of HFM with minimal headspace. Error bars represent triplicate measurements. Samples were shaken at 200 rpm, and the solution pH was roughly 6.5. Zero values indicate TCE concentrations less than the limit of detection (<1 μg/L TCE). (**b**) Pseudo-first-order fit of TCE degradation data by functionalized HFMs. Near-zero values under the limit of detection (<1 μg/L TCE) were not included in the PFO fit for 40 °C samples. (**c**) SEM image of functionalized membrane after 90 min of TCE degradation.

Below the LCST (23 °C), roughly 65% of TCE was degraded within 90 min of reaction time, while above the LCST (40 °C), 96% degradation was observed within the first 30 min (Figure 10a). Similar to MO, the primary reduction in TCE concentration was assumed to occur primarily due to catalytic degradation. A preliminary experiment utilizing PNIPAm-PMMA-3M membranes (similar weight to experimental) with oxidized NPs (thus inactive) showed a small decrease (<5%) in TCE concentration (5 mg/L) over 90 min of shaking. Degradation of TCE was further confirmed to be occurring via analysis of the MS spectrum of the contaminated solution at t = 0 min and t = 30 min (Figure S17), indicating a higher chloroethylene signal (~57.0 *m*/*z*) at t = 30 min, compared to DCE and TCE (~95 and 130 *m*/*z*, respectively), than that of the initial sample at t = 0 min.

Furthermore, this data was fitted to a PFO model (Figure 10b), yielding observed reaction rate constants of 0.0115 and 0.0993 min^−1^ at 23 °C and 40 °C, respectively. Unlike with MO, TCE degradation from these reactive membranes exhibited a stronger fit to the PFO model (R^2^ > 0.96), indicating the validity of the model for demonstrating this process. Additionally, it is important to note that the TCE concentration utilized in these experiments was 0.5 mg/L. While technologies to remediate highly contaminated water (>5 mg/L TCE) are important, systems that can remediate trace-contaminated sources (0.1–1 mg/L TCE) to be under the maximum contaminant level (MCL) of TCE (5 μg/L) [61] are vital to ensuring the long-term health of the effected communities by eliminating chronic TCE exposure. With an analytic limit of detection of 1 μg/L, the ability of the catalytic HFM systems in this research to reach below the MCL was observed above PNIPAm’s LCST.

The observed constants were corrected with respect to temperature; thus, the resulting surface-area-normalized constants were found to be 0.5251 and 2.7293 LMH for 23 °C and 40 °C, respectively (Table 4). This 420% increase (after temperature correction) can be attributed to PNIPAm’s expressed hydrophobic domains above the LCST and overall higher rates of mass transfer between the contaminant and active site. This difference between MO and TCE degradation increases above the LCST (30% and 420%, respectively) and can most likely be attributed to the difference in molecule size. The significantly higher bulk diffusion coefficient of TCE previously mentioned (Figure S15) can also be attributed, indicating high desorption rates of degradation products from the NP surface and lower mass transfer limitations overall for TCE compared to MO. This difference also indicates the limitations of utilizing MO to model catalytic systems with the intended application of TCE remediation. Additionally, it is important to note that the functionalized membrane was found to be relatively unchanged (compared to t = 0 min) via SEM analysis after 90 min of TCE degradation (Figure 10c). Unlike the membrane after MO degradation, this sample showed little surface fouling, with the observed spherical NPs remaining prominent, indicating this system’s potential for long-term TCE remediation.

Overall, incorporating PNIPAm hydrogels into catalytic HFMs shows valid advances in increasing the TCE degradation capabilities of such systems, which have not been previously reported in the literature, leading to more effective remediation techniques at environmentally relevant contaminant concentrations.

It is important to note that further methods can be utilized to optimize water remediation from TCE and other VOCs. While hydrophilic membranes offer lower operating pressures for convective water flow through traditional filtration, this work implemented this functionalization process on a primarily hydrophobic HFM. The main benefit of the membrane hydrophobicity is the ability to utilize membrane air-stripping (MAS) [62,63], which has been proven to be effective for TCE stripping (in vapor form) from liquid water sources with polypropylene HFMs [64]. A preliminary experiment was conducted with a full-sized 3M-HFM module for TCE stripping from a 200 mL solution (initial [TCE] = 3.7 ± 0.2 mg/L), utilizing a MAS set-up (Figure S18). While the control solution only dropped to 3.4 ± 0.1 mg/L TCE, the MAS solution dropped to below the limit of detection (<1 μg/L TCE) after 60 min of recycle flow, indicating that the 3M HFM’s hydrophobicity allowed for TCE to be stripped out of the contaminated solution in vapor form. By combining MAS and Fe/Pd-functionalized HFMs, even higher degradation efficiencies could be achieved, as degrading TCE in a vapor form can allow for lower mass transfer limitations between the contaminant and active site, while the water vapor can maintain adequate hydration for ZVI’s hydrogen gas generation. This process can also separate TCE from other natural organics (present in real groundwater) that could inhibit/foul the catalyst surface or act as active site competition. In this work, functionalization of the lumen side is not a significant concern, as primary degradation is desired to occur while TCE is in vapor form (present in pores and the shell side). Overall, the selection of membrane material in this work allows for potential MAS applications to achieve higher environmental remediation efficiencies in the long-term.

## 4. Conclusions

This research investigated the integration of the temperature-responsive polymer, PNIPAm, into Fe/Pd-PMMA-functionalized hollow fiber membranes, establishing a significant step towards the scalability of these systems. Temperature-responsive functionalization of HFMs was achieved, a synthesis aspect that has not been previously reported in the literature. After functionalization, the dip-coated flat sheet and HFMs showed weight gains of roughly 9% and 15%, respectively, indicating significant retention of stimuli-responsive hydrogel. Due to enhanced hydrophobic interactions between the hydrogel matrix and the pollutant (expressed at T > LCST_PNIPAm_), degradation increases of 30% and 420% for azo dyes (methyl orange) and VOCs (trichloroethylene), respectively, were observed with the developed HFM in batch mode, overcoming previously-reported mass transfer limitations that occur when integrating such catalysts into polymer-membrane matrixes. These findings signified significant novelties in the remediation field, as the range of contaminants that such membrane systems can treat in groundwater sources has been expanded to MO and TCE, both previously unreported in the literature with these thermoresponsive systems. Furthermore, the resulting degradation data was modeled using a pseudo-first-order model, obtaining surface-area and temperature-corrected reaction rate constants of 0.6247 and 0.5251 at 23 °C and 0.8147 and 2.7293 at 40 °C for methyl orange and TCE, respectively. Overall, the implementation of catalytic and temperature-responsive functionalizations into hollow fiber membrane systems with superior surface area per packing volume provided novel advances to the field of membrane-based water remediation. This work demonstrated the capabilities of developing temperature-responsive membrane modules that can result in treating larger volumes of contaminated water per unit time than current established filtration methods.

In future work, the effect of natural organic presence can be quantified in relation to degradation efficiency over long periods of treatment time, thus simulating more realistic remediation scenarios. The ability of catalytic hollow fiber membranes to degrade VOCs in a convective mode can be further studied as well, which could prove its efficacy in at-home water treatment modules with low replacement needs. Additionally, a grafting method could be established for attaching the polymer/catalyst system to the membrane domain, furthering the long-term stability and low metal leaching of the resulting filter.

## Figures and Tables

**Table 1:** Membrane characteristics of flat sheet and hollow fiber membranes utilized in this study.

	PVDF400-Flat Sheet	PVDF650-Flat Sheet	Tribore-HFM	3M-HFM	Lifestraw-HFM
Material	Hydrophilized polyvinylidene fluoride (PVDF)	Hydrophilized polyvinylidene fluoride (PVDF)	Hydrophobic polyvinylidene fluoride (PVDF) *^1^	Hydrophobic polypropylene (PP) *^1^	Hydrophilized polyether sulfone (hPES) *^1^
Mean pore size	40.6 nm, with the largest being 300–400 nm (SEM)	62.2 nm, with the largest being 300–400 nm (SEM)	1153 nm	40 nm (manufacturer)	200 nm (manufacturer)
Thickness	178 ± 13 μm	178 ± 13 μm	412.6 ± 106.2 μm *^1^	41.8 ± 2.2 μm	85.5 ± 3.5 μm
Bulk porosity	46% *^2^	47%	24% *^1^	24% *^1^ or 40% (manufacturer)	45% *^1^
Shell surface mean pore size	Not applicable	Not applicable	57 ± 35 nm *^1^	42 ± 17 nm *^1^	140 ± 87 nm *^1^
Lumen surface mean pore size	Not applicable	Not applicable	1346 ± 1086 *^1^	46 ± 27 nm *^1^	748 ± 896 nm *^1^
Tortuosity	Not measured	Not measured	3.1692 *^1^	Not measured	Not measured

*^1^ Data obtained from Baldridge et al. [35]. *^2^ Data obtained from a prior study (Mills et al.) [36].

**Table 2:** Observed and surface area-normalized reaction rate constants (k_obs_ and k_sa_) of batch-mode MO degradation via Fe/Pd NPs in solution. The average diameter of NPs utilized for calculations was 36 nm on average.

T (°C)	k_obs_ (1/min)	ρ_m_ (g/L)	a_s_ (m^2^/g)	k_sa_ (LMH)	Temperature-Normalized k_sa_ (LMH) *
22.5 °C	0.0208	5	21.2	0.0118	0.0118
50 °C	0.0424	5	21.2	0.0240	0.0108

* Normalized with respect to 22.0 °C using the Arrhenius equation and an activation energy of ~23 kJ/mol from the ZVI system of Shih et al. [55].

**Table 3:** Observed and surface area-normalized reaction rate constants (k_obs_ and k_sa_) of batch-mode MO degradation via Fe/Pd-PNIPAm-PMMA-functionalized HFMs in solution. The average diameter of NPs utilized for calculations was 34.8 nm on average.

T (°C)	k_obs_ (1/min)	ρ_m_ (g/L)	a_s_ (m^2^/g)	k_sa_ (LMH)	Temperature-Normalized k_sa_ (LMH) *
23 °C	0.0114	0.05	21.9	0.6247	0.6247
40 °C	0.0247	0.05	21.9	1.3534	0.8147

* Normalized with respect to 23.0 °C using the Arrhenius equation and an activation energy of ~23 kJ/mol from the ZVI system of Shih et al. [55].

**Table 4:** Observed and surface area-normalized reaction rate constants (k_obs_ and k_sa_) of batch-mode TCE degradation via Fe/Pd-PNIPAm-PMMA-functionalized HFMs in solution. The average diameter of NPs utilized for calculations was 34.8 nm on average.

T (°C)	k_obs_ (1/min)	ρ_m_ (g/L)	a_s_ (m^2^/g)	k_sa_ (LMH)	Temperature-Normalized k_sa_ (LMH) *
23 °C	0.0115	0.06	21.9	0.5251	0.5251
40 °C	0.0993	0.06	21.9	4.5342	2.7293

* Normalized with respect to 22.0 °C using the Arrhenius equation and an activation energy of ~23 kJ/mol from the ZVI system of Shih et al. [55].

## Data Availability

The data that support the findings of this study are available from the corresponding author upon reasonable request.

## Supplementary Materials

The following supporting information can be downloaded at: https://www.mdpi.com/article/10.3390/nano13142041/s1, Section A. Figure S1: Calibration curve of known TCE concentration (after 100:1 injection dilution) versus the area under the measured peak via GCMS analysis. Hexane (GCMS grade) was utilized as the solvent; Figure S2: GCMS analysis of TCE standards. (a) Total ion chromatograph (TIC) of 1 mg/L TCE solution (after 100:1 injection dilution) and (b) MS spectrum results (counts vs. mass-to-charge) for TCE peak displayed; Figure S3: Volumetric water flux versus applied pressure of flat sheet PVDF400 and PVDF650 commercial membranes; Figure S4: Full-thickness cross-section SEM image of an asymmetric PVDF400 membrane; Figure S5: SEM of cross-section 3M-HFM with corresponding schematic of hollow fiber membrane structure, shell side, and lumen side; Figure S6: FTIR of unfunctionalized and functionalized membrane samples; Figure S7: SEM image of microfibers holding together 3M-HFM; Figure S8: Fe/Pd particle size of functionalized (a) PVDF650 and (b) 3M-HFM; Figure S9: Cross-section SEM image of cross-section pores of 3M-HFM functionalized with Fe/Pd bimetallic nanoparticles, PNIPAm, and PMMA; Figure S10: Schematic of Fe/Pd NP synthesis in solution; Figure S11: An XRD diffractogram of (a) freshly made metallic iron (Fe^0^) particles, (b) freshly made palladium particles, (c) palladium coated iron particles, (d) deliberately oxidized Fe/Pd particles, (e) regenerated Fe/Pd particles, and (f) Fe_2_O_3_ particles [46]; Figure S12: TEM, EDX, EELS analysis of Fe/Pd NPs free in solution; Figure S13: Size distribution of Fe/Pd NPs free in solution; Figure S14: Calibration curve of MO standards, analyzed using UV-Vis; Figure S15: Calculated bulk diffusion coefficients for MO, TCE, and PCB-1 water contaminants in a water solvent, with respect to temperature; Figure S16: TEM and EEL analysis of Fe/Pd NPs stored in DIW solutions with different dissolved oxygen (DO) concentrations over a 24 h period; Figure S17: GCMS analysis of contaminant solution during PNIPAm/PMMA-Fe/Pd-functionalized HFM experimentation above the LCST of PNIPAm; Figure S18: Schematic of MAS set-up utilized for TCE stripping; Section B. Table S1: EELS analysis of Fe/Pd nanoparticle. Section C. Supplementary Methods.
