# Supplementary material for: Enhanced Degradation of Methyl Orange and Trichloroethylene with PNIPAm-PMMA-Fe/Pd-Functionalized Hollow Fiber Membranes
Source: Nanomaterials (Basel). 2023 Jul 10;13(14):2041. doi: 10.3390/nano13142041 (PMC10386459; doi:10.3390/nano13142041)
Supplement: Supplementary file 1 [file nanomaterials-13-02041-s001.zip › nanomaterials-2465432-supplementary.pdf]

nanomaterials

Supplementary Materials

Enhanced Degradation of Methyl Orange and Trichloroethylene with PNIPAm-PMMA-Fe/Pd-Functionalized Hollow Fiber Membranes

Rollie Mills, Cameron Tvrdik, Andrew Lin and Dibakar Bhattacharyya *

Department of Chemical and Materials Engineering, University of Kentucky; Lexington, KY 40508, USA; rolliegmills@uky.edu (R.M.); cameron.tvrdik@uky.edu (C.T.); andrew.lin@uky.edu (A.L.)
* Correspondence: db@uky.edu; Tel.: +1-(859)-312-7790

This Supplementary Materials consists of the following sections:

- Section A: Figures S1-S18
- Section B: Table S1
- Section C: Supplementary Methods

Section A: Supplementary Figures

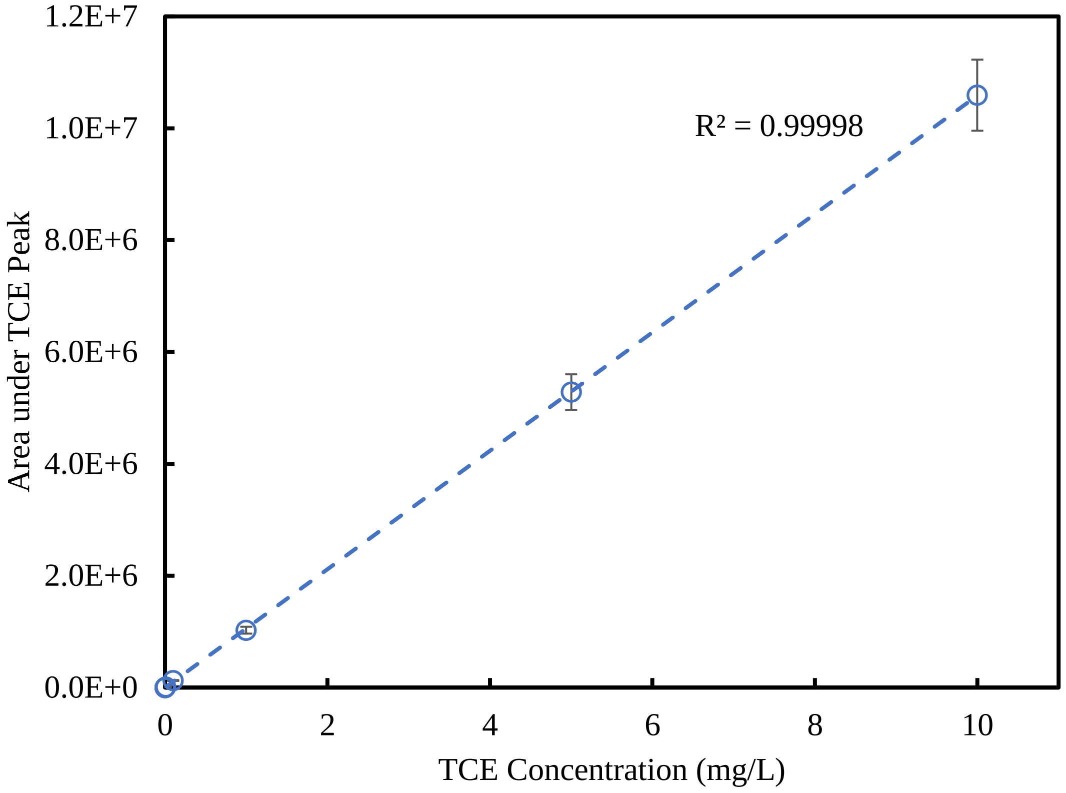

Figure S1. Calibration curve of known TCE concentration (after 100:1 injection dilution) versus the area under the measured peak via GCMS analysis. Hexane (GCMS grade) was utilized as the solvent. Error bars represent triplicate measurement error of GCMS (approximately 6%). Agilent 8890 GC system was utilized with 5977C GC/MSD.

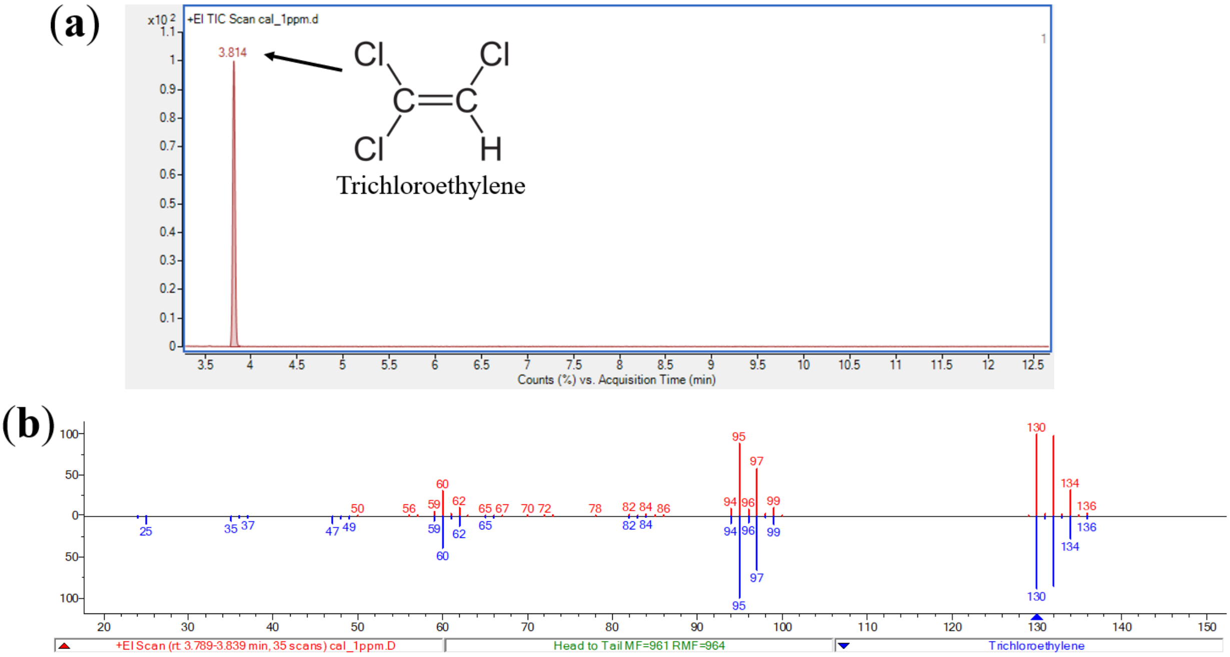

Figure S2. GCMS analysis of TCE standards. (**a**) Total ion chromatograph (TIC) of 1 mg/L TCE solution (after 100:1 injection dilution) and (**b**) MS spectrum results (counts vs. mass-to-charge) for TCE peak displayed, where the red lines signify the scanned sample and the blue lines signify the known TCE spectrum from the National Institute of Standards and Technology (NIST) library. The corresponding peak at an acquisition time of 3.814 min signifies TCE presence, confirmed with known TCE standards obtained from Sigma Aldrich. Hexane (GCMS grade) was utilized as the solvent. Agilent 8890 GC system was utilized with 5977C GC/MSD.

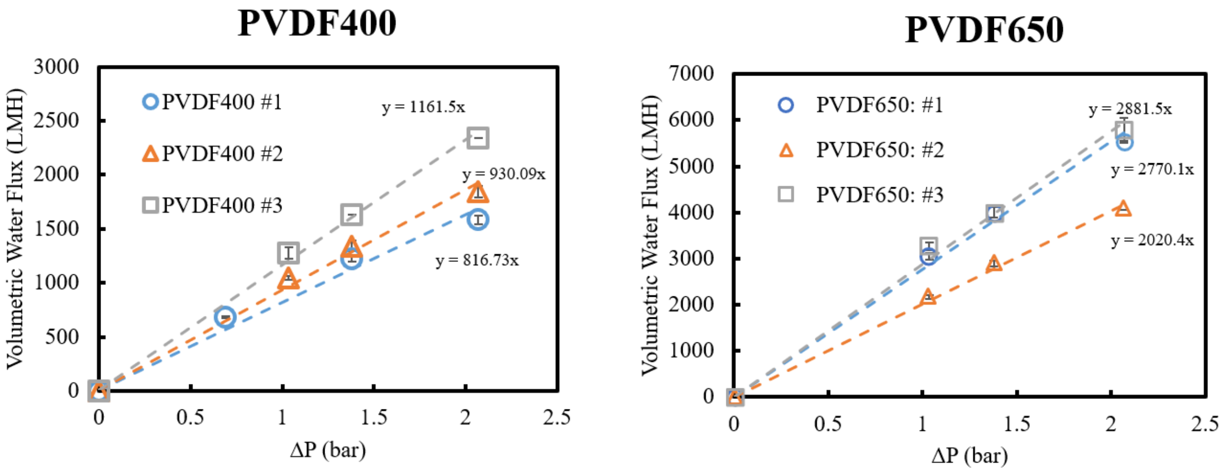

Figure S3. Volumetric water flux versus applied pressure of flat-sheet PVDF400 and PVDF650 commercial membranes. Error bars indicate triplicate measurements on each individual membrane. Each linear line represents a different sample of membrane tested. .

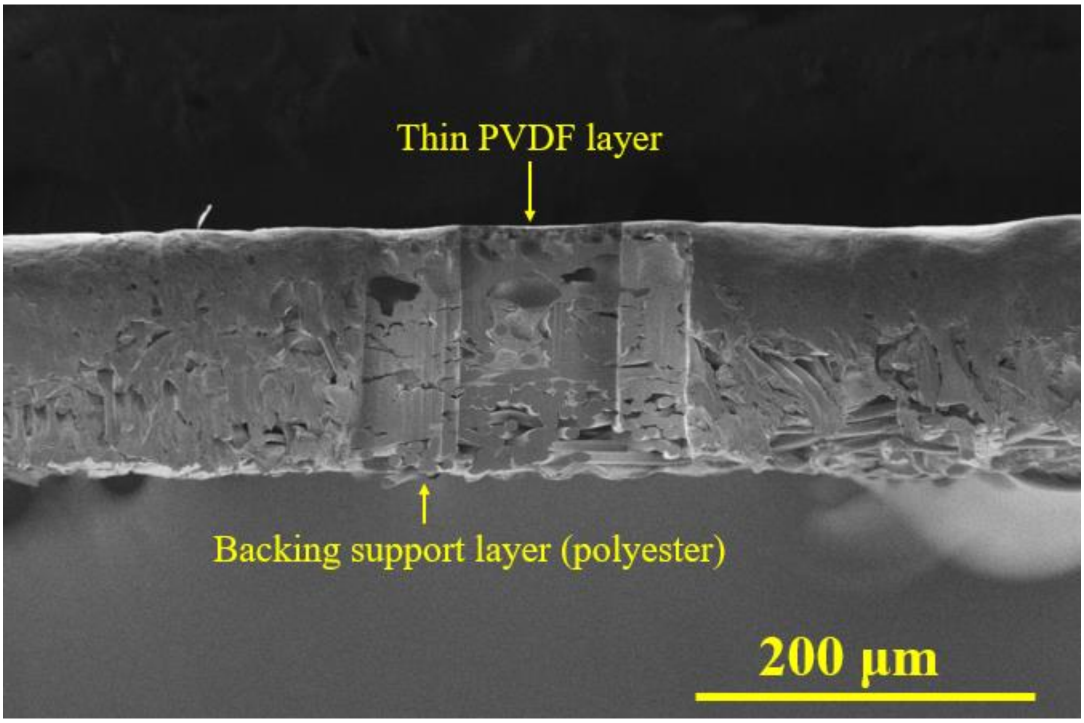

Figure S4. Full-thickness cross-section SEM image of an asymmetric PVDF400 membrane. This membrane, as well as the PVDF650, consists of a thin PVDF separating layer and an open backing support (made from polyester).

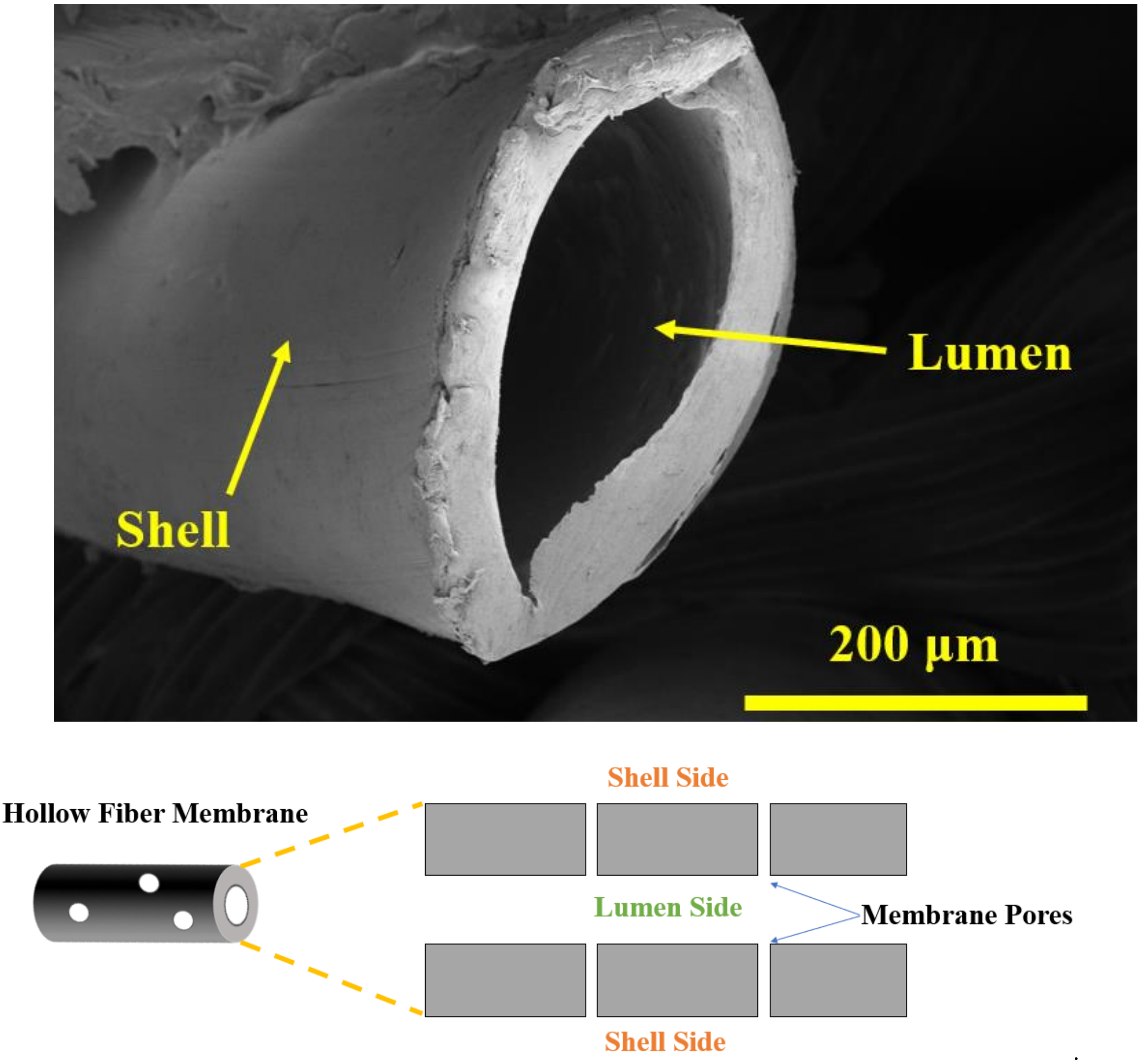

Figure S5. SEM of cross-section 3M-HFM with corresponding schematic of hollow fiber membrane structure, shell side, and lumen side. Note that pores depicted on schematic are not an accurate representation of the 3M HFM pores, as they are created using thermal stretching.

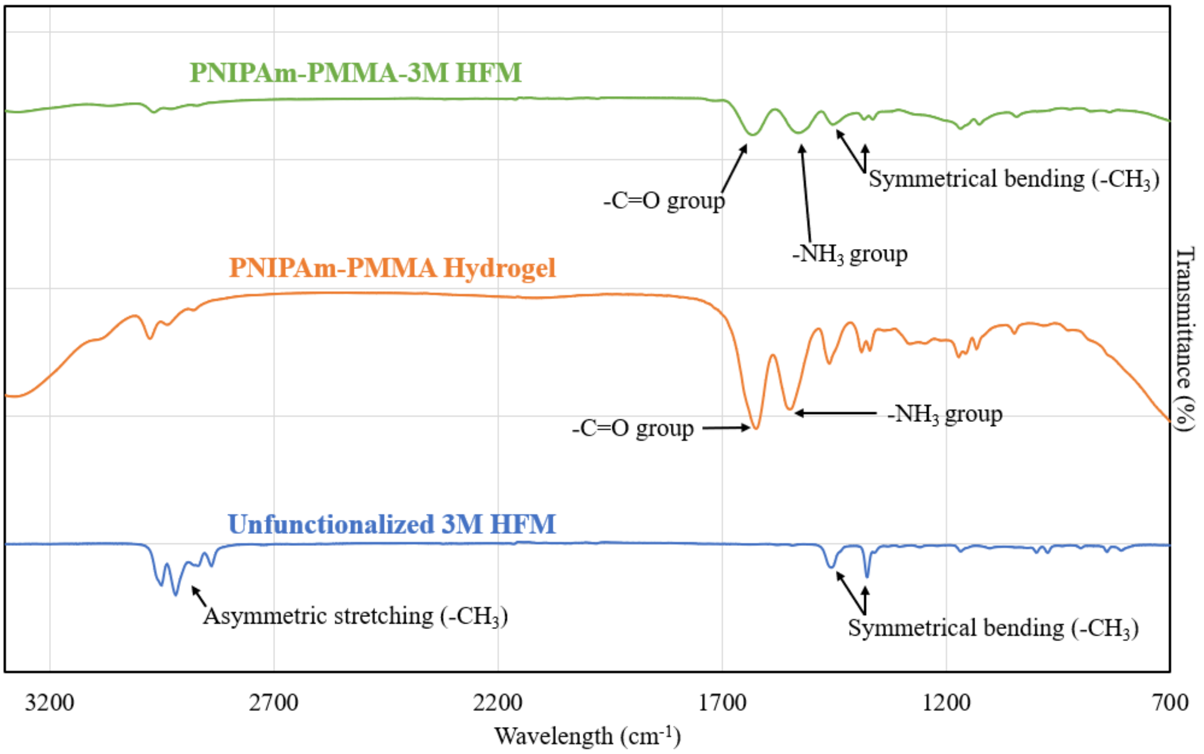

Figure S6. FTIR of unfunctionalized and functionalized membrane samples. PNIPAm-PMMA's -NH_3 group (~1540 cm^{-1}) and -C=O group (~1650 cm^{-1}) was utilized to confirm functionalization of 3M HFM.

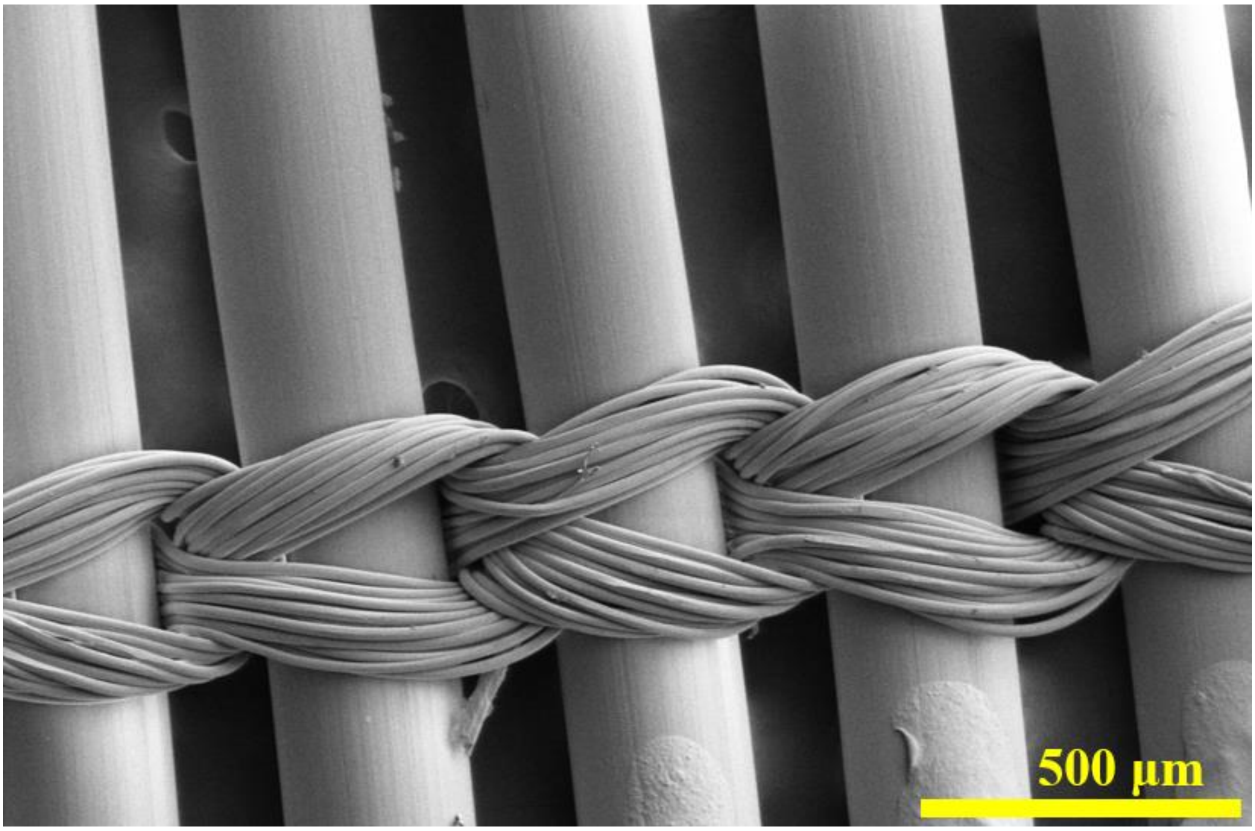

Figure S7. SEM image of microfibers holding together 3M-HFM.

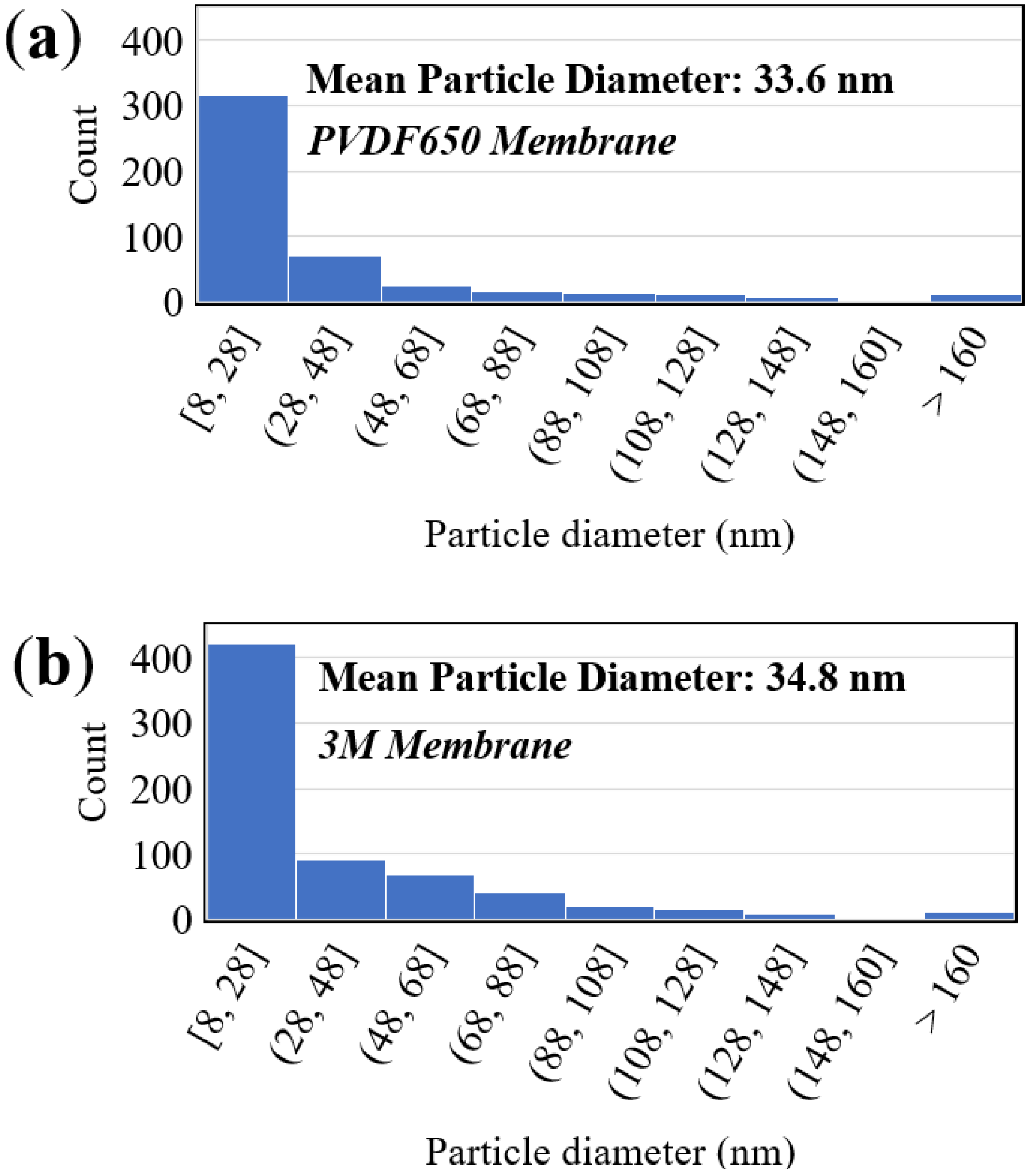

Figure S8. Fe/Pd particle size of functionalized (**a**) PVDF650 and (**b**) 3M-HFM. SEM images were analyzed using ImageJ to produce size estimations.

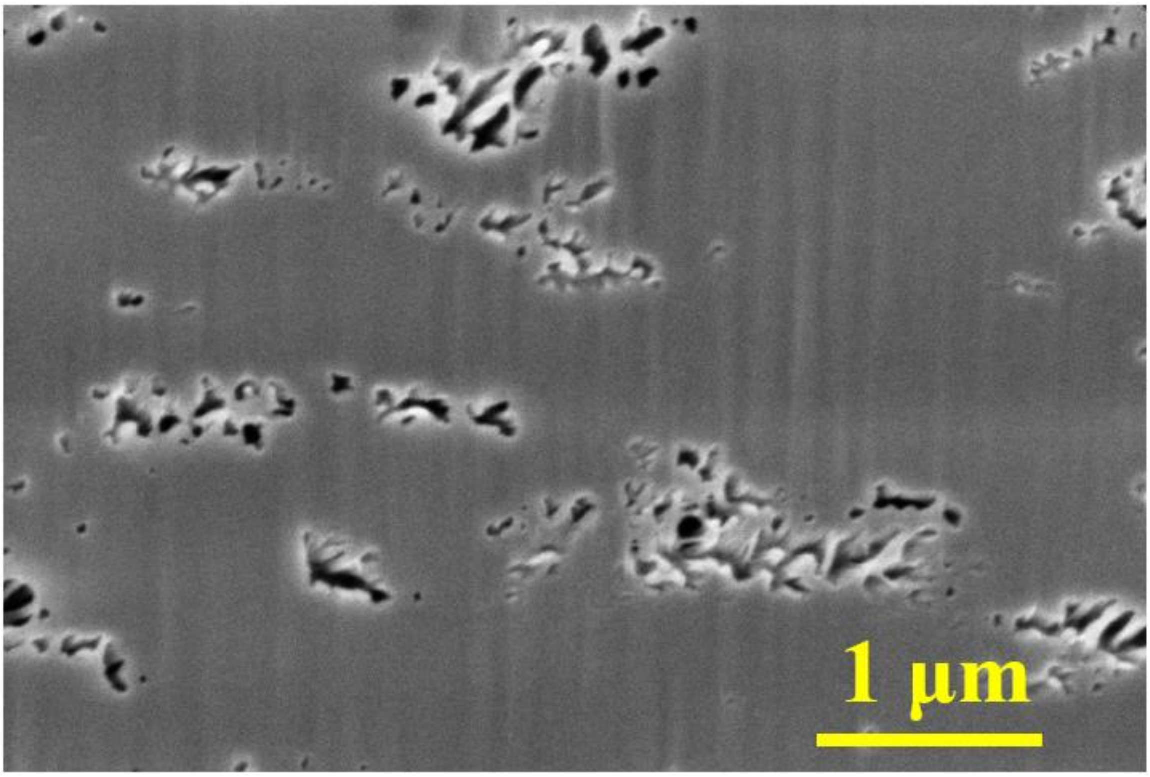

Figure S9. Cross-section SEM image of cross-section pores of 3M-HFM functionalized with Fe/Pd bimetallic nanoparticles, PNIPAm, and PMMA.

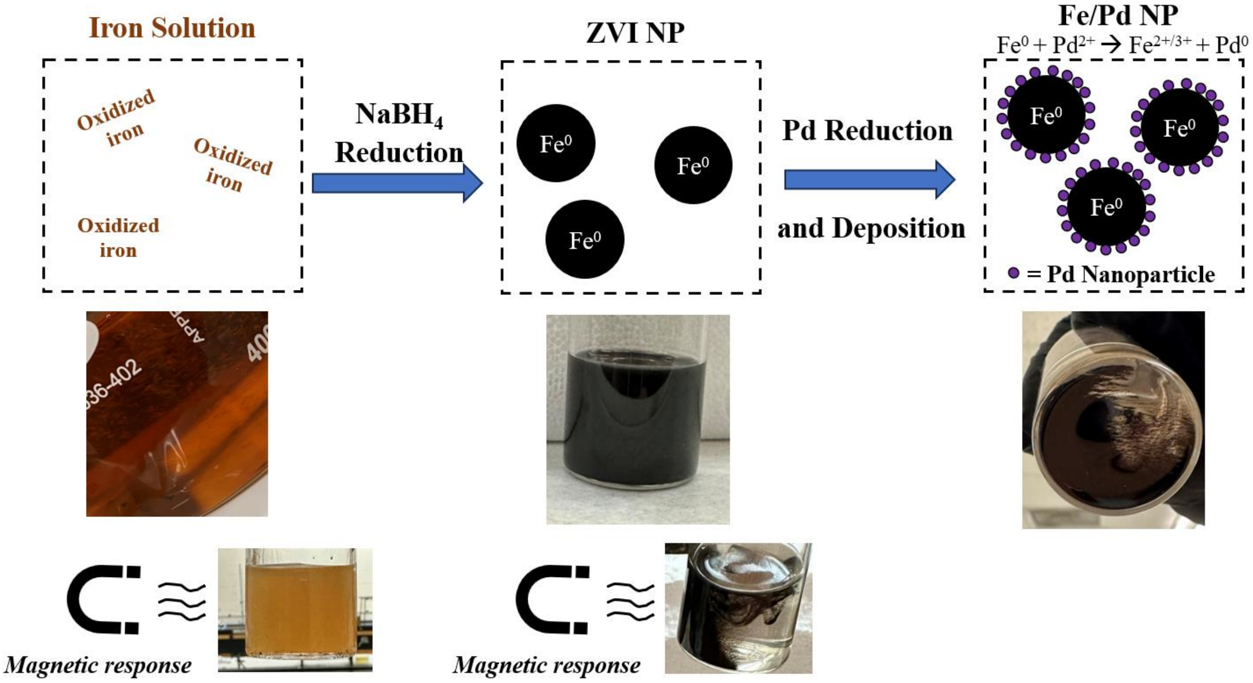

Figure S10. Schematic of Fe/Pd NP synthesis in solution. Highlighted in the schematic is color change of NPs, as well as magnetic response, from iron oxide to reduced ZVI.

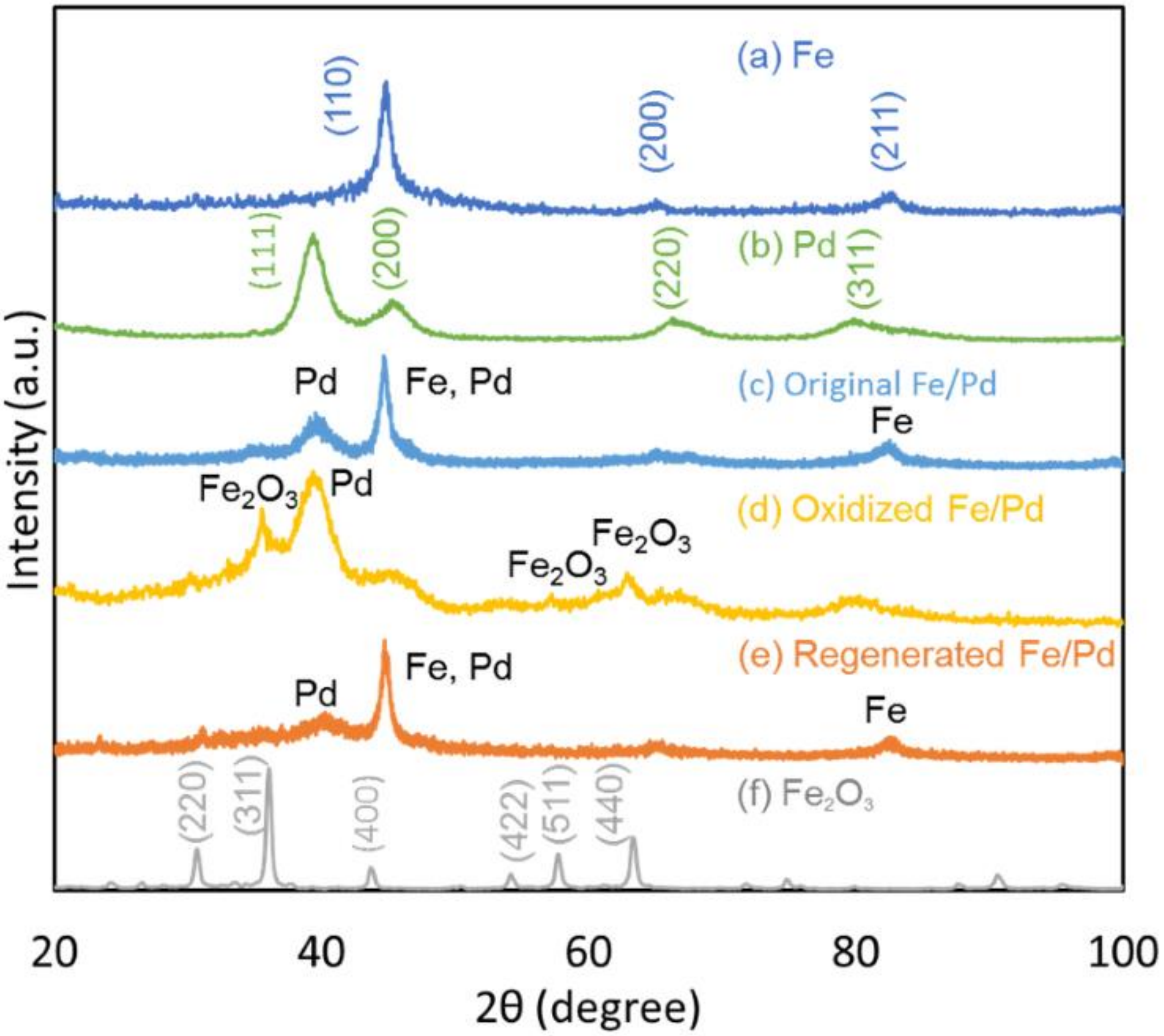

Figure S11. An XRD diffractogram of (**a**) freshly made metallic iron (Fe^0) particles, (**b**) freshly made palladium particles, (**c**) palladium coated iron particles, (**d**) deliberately oxidized Fe/Pd particles, (**e**) regenerated Fe/Pd particles and (**f**) Fe2O3 particles (Sigma Aldrich). Reproduced with permission from: Wan, H., Bhattacharyya, D., et al. Pore Functionalized PVDF Membranes with In-Situ Synthesized Metal Nanoparticles: Material Characterization, and Toxic Organic Degradation. *J Memb Sci* **2017**, *530*, 147-157, doi:10.1016/j.memsci.2017.02.021.

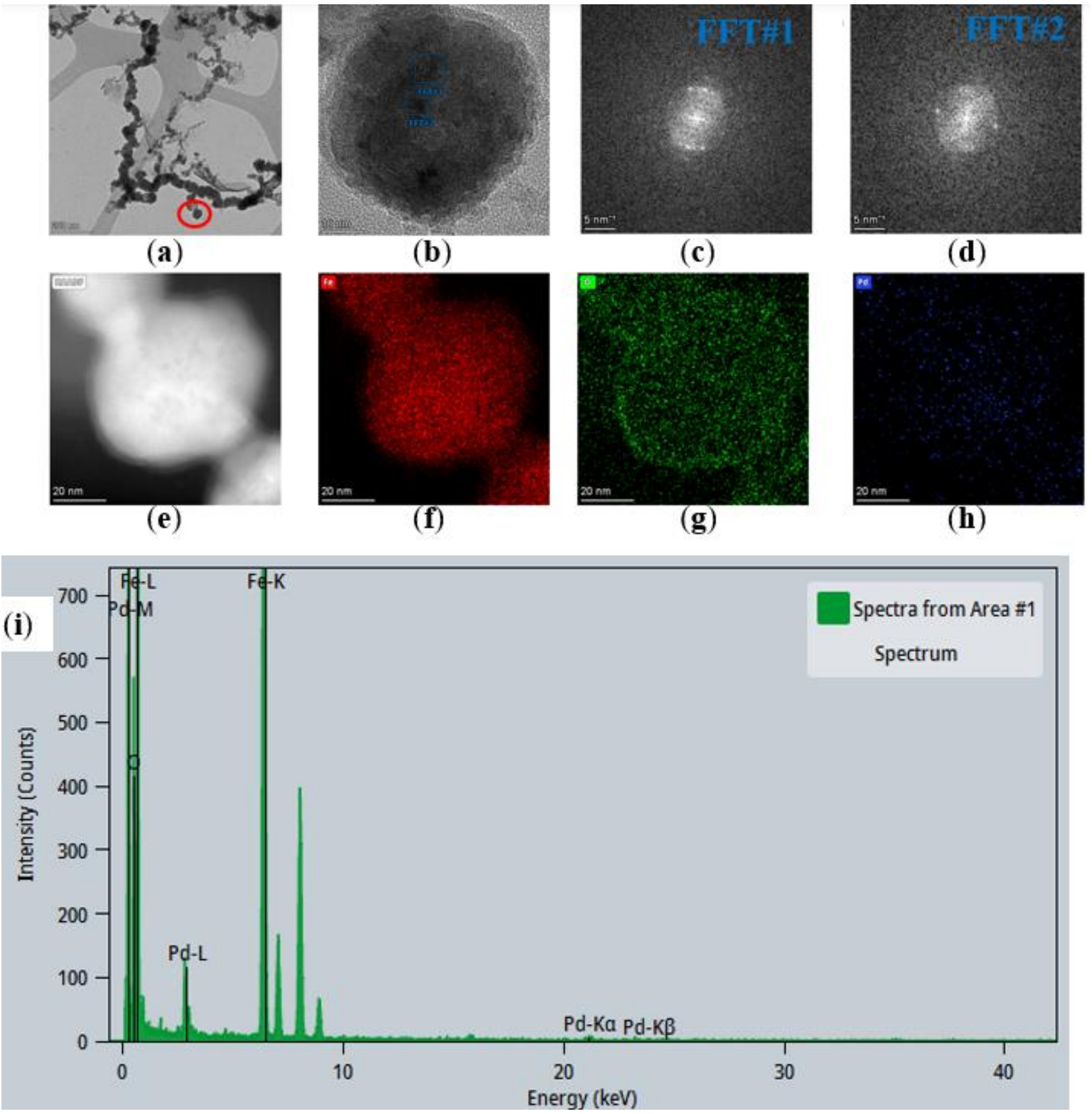

Figure S12. TEM, EDX, EELS analysis of Fe/Pd NPs free in solution. (**a**) Low-magnification TEM image of Fe/Pd NP chains with (**b**) high-magnification TEM image of a single particle. Image (**c**) and (**d**) are Fast-Fourier Transforms (FFT) of Region #1 and Region #2, respectively. A high-angle annular dark-field imaging (HAADF) image (**e**) of a NP is present with EELS analysis for (**f**) iron, (**g**) oxygen, and (**h**) palladium, respectively. An EELS spectra is provided (**i**) when analyzing the area of a single NP.

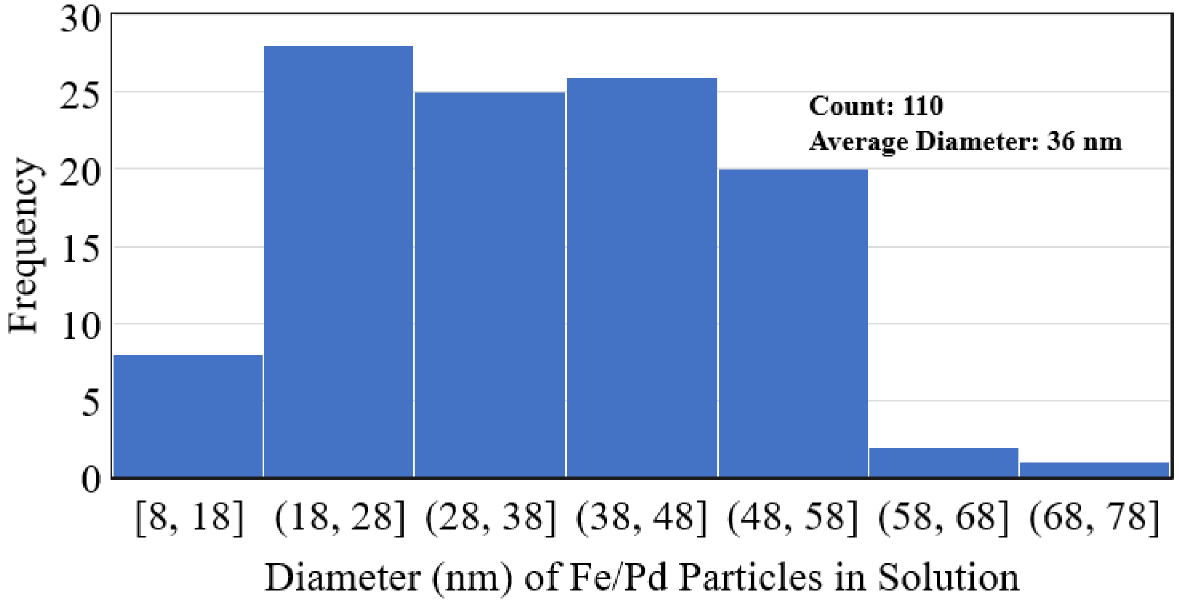

Figure S13. Size distribution of Fe/Pd NPs free in solution. The average diameter of the NPs was roughly 36 nm.

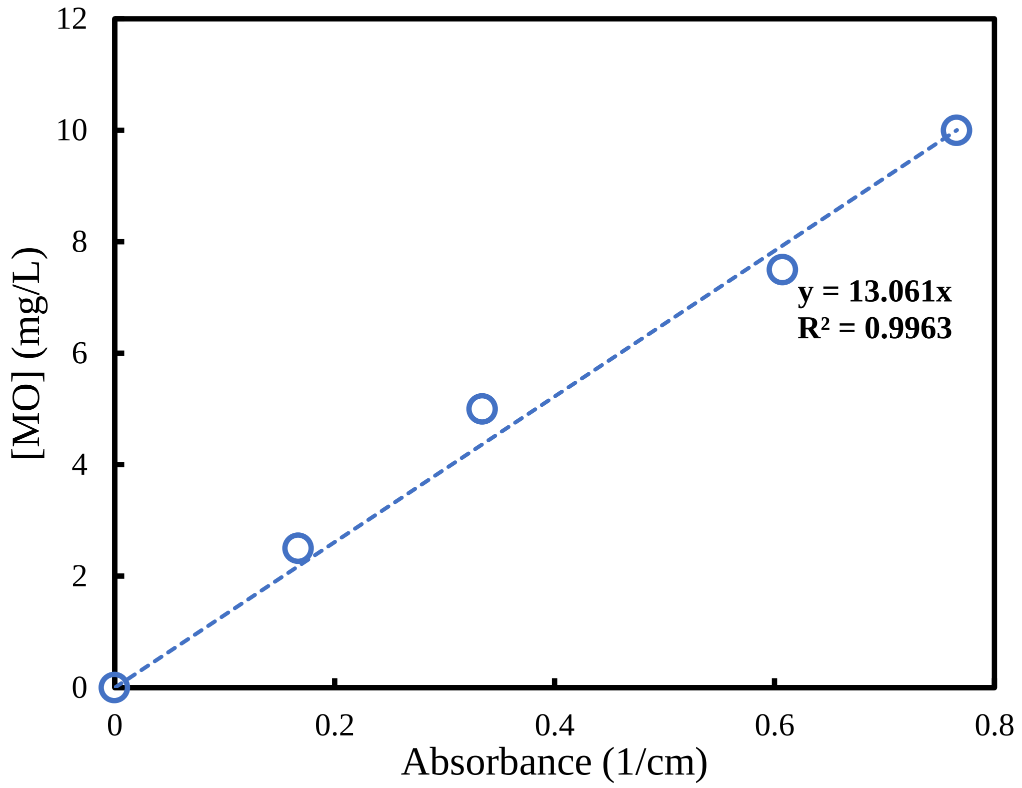

Figure S14. Calibration curve of MO standards, analyzed using UV-Vis. Absorbance values measured at wavelength of 464 nm, which indicates the N=N bond of MO.

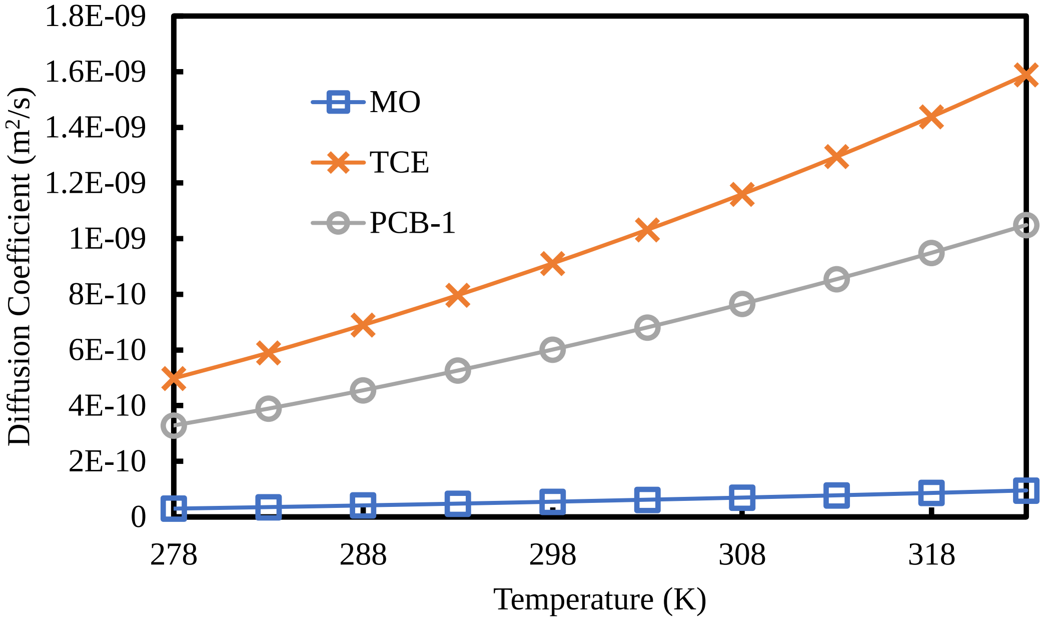

Figure S15. Calculated bulk diffusion coefficients for MO, TCE, and PCB-1 water contaminants in a water solvent, with respect to temperature. The Stokes-Einstein equation was utilized for modelling. Viscosity values were normalized with respect to changes in temperature. The radius of each molecule was estimated, based on geometry and chemical bonds.

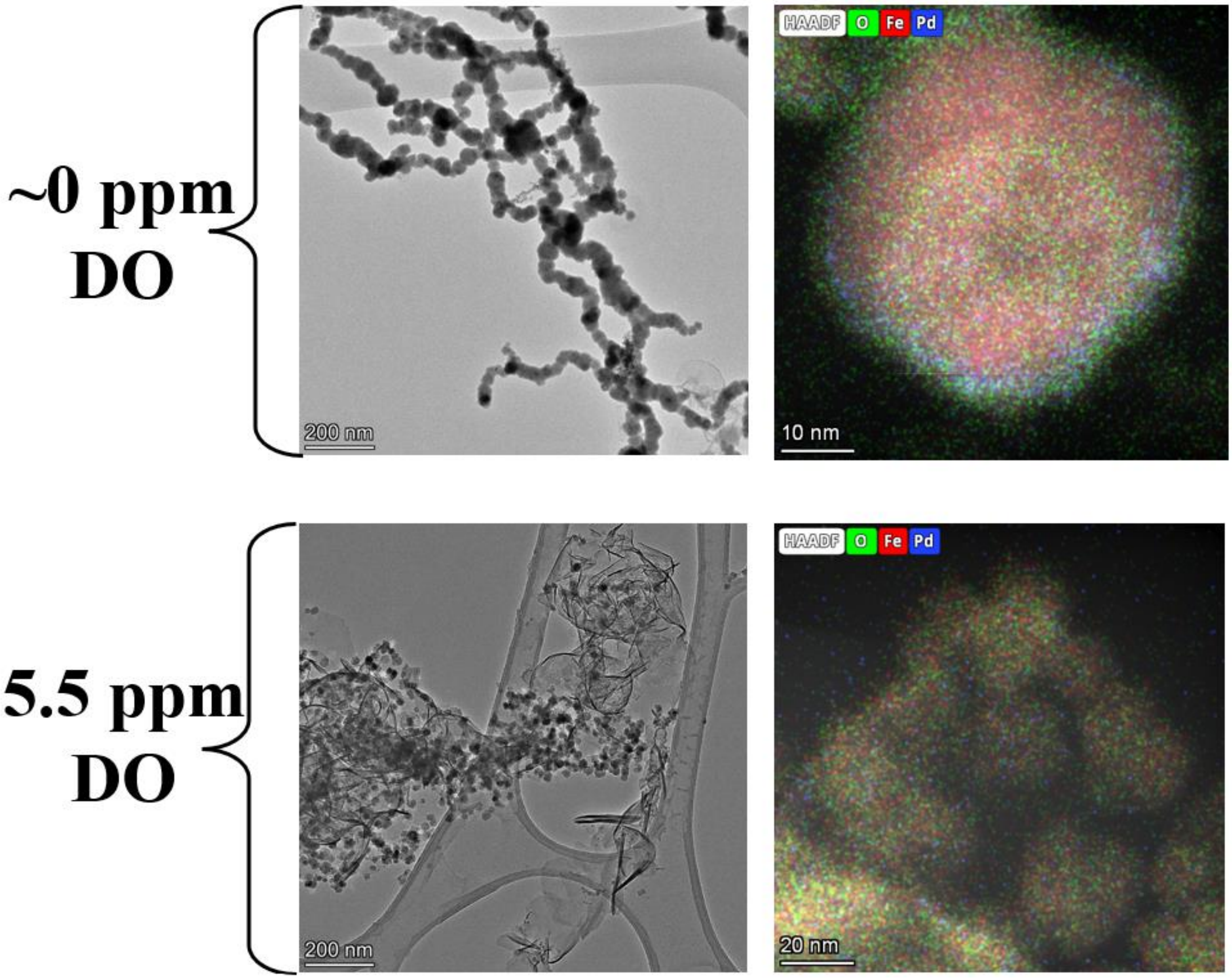

Figure S16. TEM and EEL analysis of Fe/Pd NPs stored in DIW solutions with different dissolved oxygen (DO) concentrations over a 24-hour period. DO concentration of water was altered by bubbling O_2 (increase concentration) or N_2 (decrease concentration) until desired DO was reached. DO concentration was measured with a VWR OX 4100L probe.

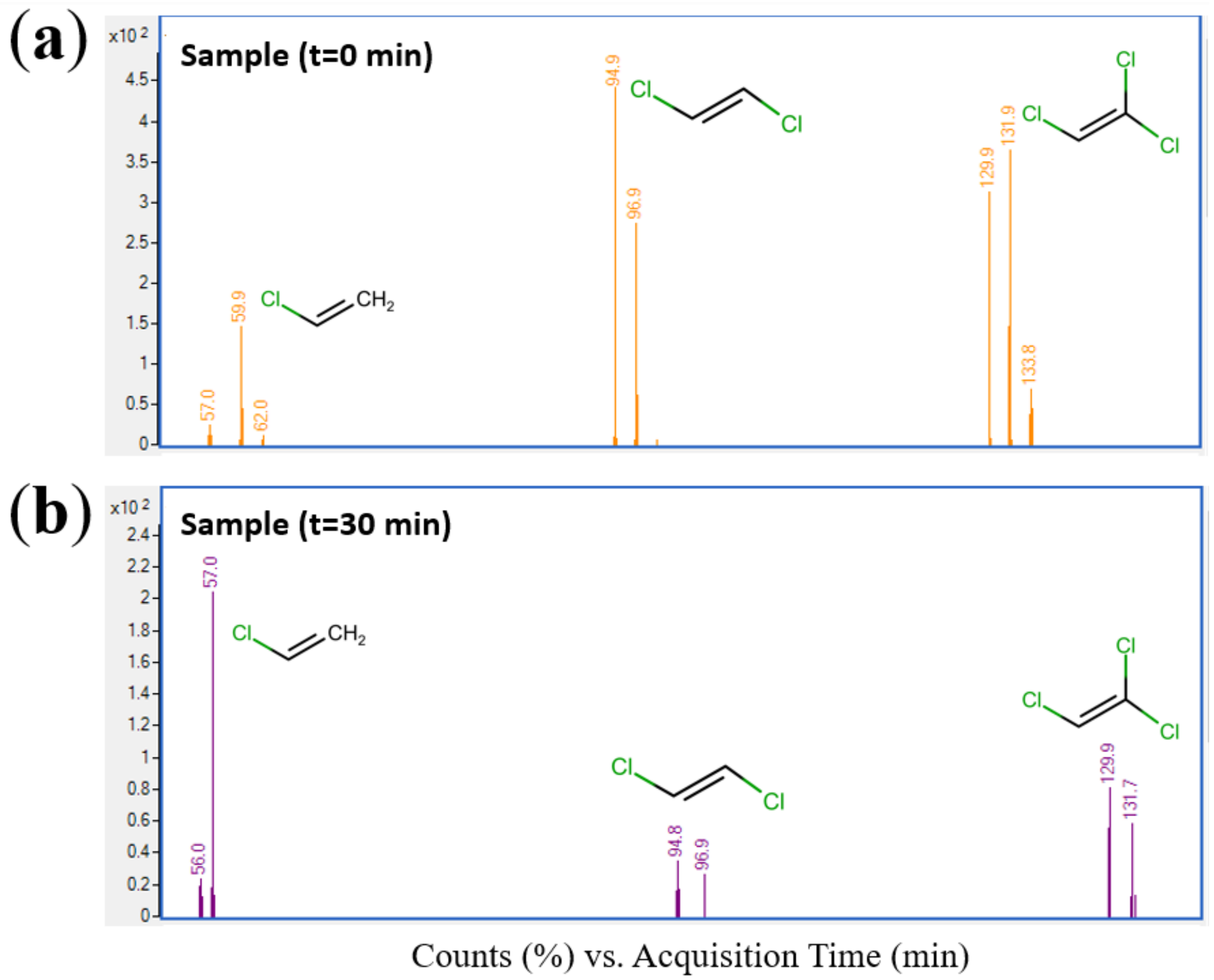

Figure S17. GCMS analysis of contaminant solution during PNIPAm/PMMA-Fe/Pd-functionalized HFM experimentation above the LCST of PNIPAm. MS spectrum of solution at (**a**) t=0 min and (**b**) t=30 min. Both points had peak acquisition time of ~3.8 min, which agrees with TCE standard. Aldrich. Hexane (GCMS grade) was utilized as the solvent. Agilent 8890 GC system was utilized with 5977C GC/MSD.

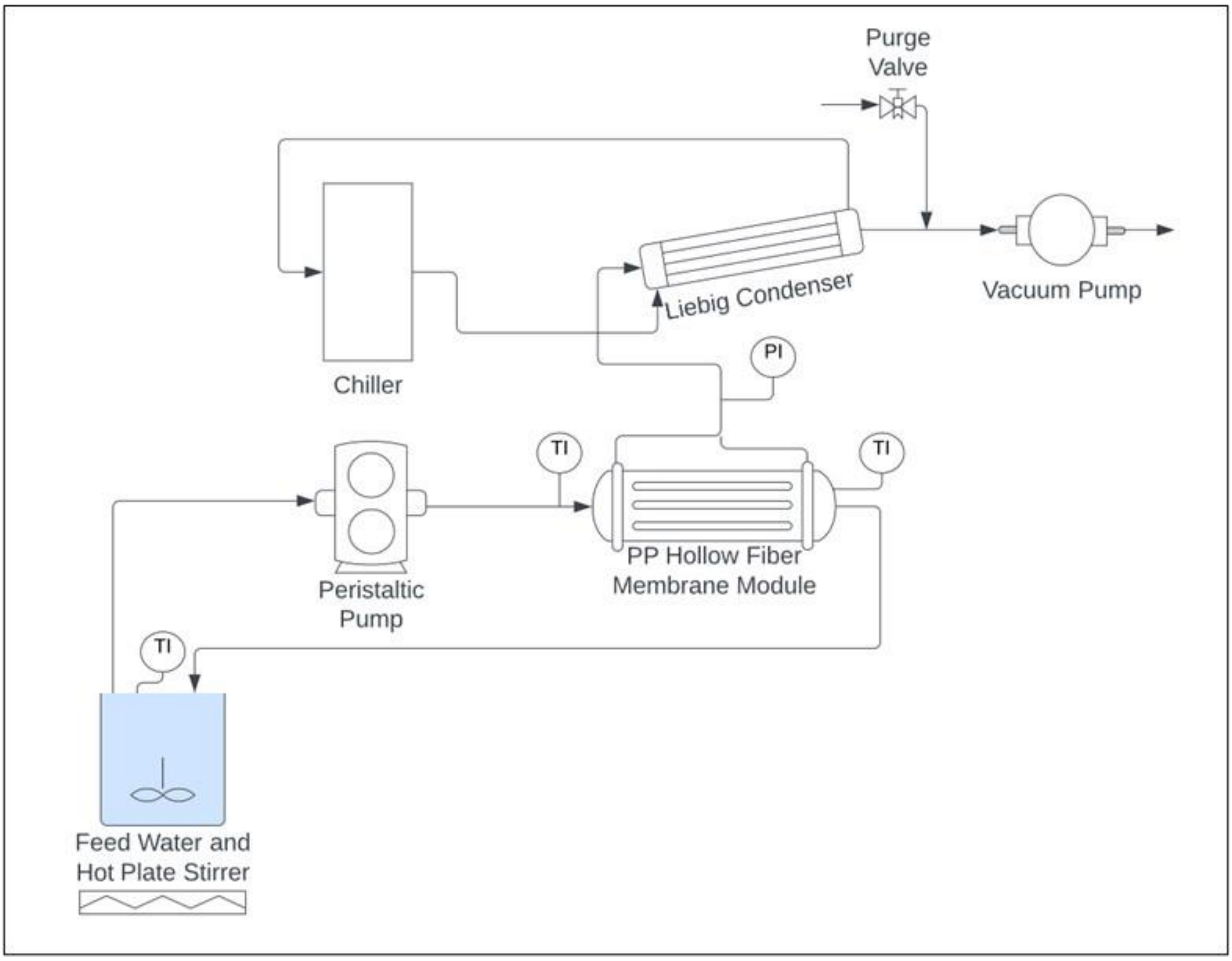

Figure S18. Schematic of MAS set-up utilized for TCE stripping. Membrane used was a 3M HFM module. Initial TCE concentration was ~3.7 mg/L and solution volume was 200 mL.

Section B: Supplementary Tables

Table S1. EELS analysis of a Fe/Pd nanoparticle.

Z	Element	Family	Atomic Fraction (%)	Atomic Error (%)	Mass Fraction (%)	Mass Error (%)	Fit error (%)
8	O	K	33.43	5.81	12.11	1.3	0.7
26	Fe	K	63.34	13.88	80.11	13.72	1.08
46	Pd	L	3.23	0.68	7.77	1.23	0.62

.

Section C: Supplementary Methods

Transmission electron microscopy (TEM), Scanning electron microscopy (SEM) and energy dispersive X-ray (EDX)

To analyze Fe/Pd nanoparticles with transmission electron microscopy (TEM), the NP solution was sonicated for 5 minutes to avoid agglomeration on the TEM grid. 0.5 mL of the solution was then added to 19.5 mL ethanol, and sonicated again for 5 minutes. A TEM grid was held by self-closing tweezers and dipped into the solution for 5 seconds. The grid was then heated in a vacuum oven overnight at 60°C. TEM grids were purchased from Ted Pella (Lacey Carbon, 300 mesh, Copper, approximate grid hole size: 63µm). The NPs were analyzed using a FEI Talos F200X transmission electron microscope.

Before SEM and EDX analysis of the membrane, samples were attached to the mounting surface using conductive carbon tape (Nishin) and 2-5 nm layer of platinum (Pt) was applied using the Leica EM ACE600 sputter coater. Electron microscopy imaging was done using the FEI Helios Scanning Electron Microscope. For cross-section imaging, broad beam ion milling was conducted (before SEM analysis) using a JEOL Cross Section Polisher (CP) for minimization of sample damage and melting.

Attenuated total reflectance Fourier transform infrared (ATR-FTIR)

Attenuated total reflectance Fourier transform infrared (ATR-FTIR) was utilized to determine presence of hydrogels functionalized onto membrane surface. Analysis was conducted using a Thermo Fisher Scientific iS50 FT-IR system. Sample transmittance was analyzed using the following settings: 32 No of scans, resolution of 8, and data spacing of 0.964 cm^{-1}. Background signal was collected before sample analysis.

Inductively coupled plasma (ICP)

For quantification of iron immobilization into the membrane-hydrogel matrix, an Agilent 7800 inductively coupled plasma mass spectrometer (ICP-MS) was utilized. An iron standard solution was purchased from VWR (EM1.70326.0100) for a calibration curve. A 1.0000 R^2 value was obtained from fitting the calibration curve with a linear fit (concentrations ranging from 0 to 100 mg/L Fe^{2+}). Triplicate samples were taken for each sample with blank rinses in between all sampling. Each sample consisted of 2% nitric acid matrices. Appropriate control samples, as well as blind iron samples, were utilized to ensure accuracy of results.
